# Supplementary material for: Epigenetic mechanisms to propagate histone acetylation by p300/CBP
Source: Nat Commun. 2023 Jul 17;14:4103. doi: 10.1038/s41467-023-39735-4 (PMC10352329; doi:10.1038/s41467-023-39735-4)
Supplement: Supplementary file 1 — Supplementary Information [file 41467_2023_39735_MOESM1_ESM.pdf]

Supplementary Information

Epigenetic mechanisms to propagate histone acetylation by p300/CBP

Masaki Kikuchi[1], Satoshi Morita[1], Masatoshi Wakamori[1], Shin Sato[1], Tomomi Uchikubo-Kamo[2], Takehiro Suzuki[3], Naoshi Dohmae[3], Mikako Shirouzu[2] & Takashi Umehara[1,*]

[1]Laboratory for Epigenetics Drug Discovery, RIKEN Center for Biosystems Dynamics Research, 1-7-22 Suehiro-cho, Tsurumi, Yokohama 230-0045, Japan.

[2]Laboratory for Protein Functional and Structural Biology, RIKEN Center for Biosystems Dynamics Research, 1-7-22 Suehiro-cho, Tsurumi, Yokohama 230-0045, Japan.

[3]Biomolecular Characterization Unit, Technology Platform Division, RIKEN Center for Sustainable Resource Science, 2-1 Hirosawa, Wako, Saitama 351-0198, Japan.

[*]Corresponding author. e-mail: takashi.umehara@riken.jp

Supplementary Figure 1

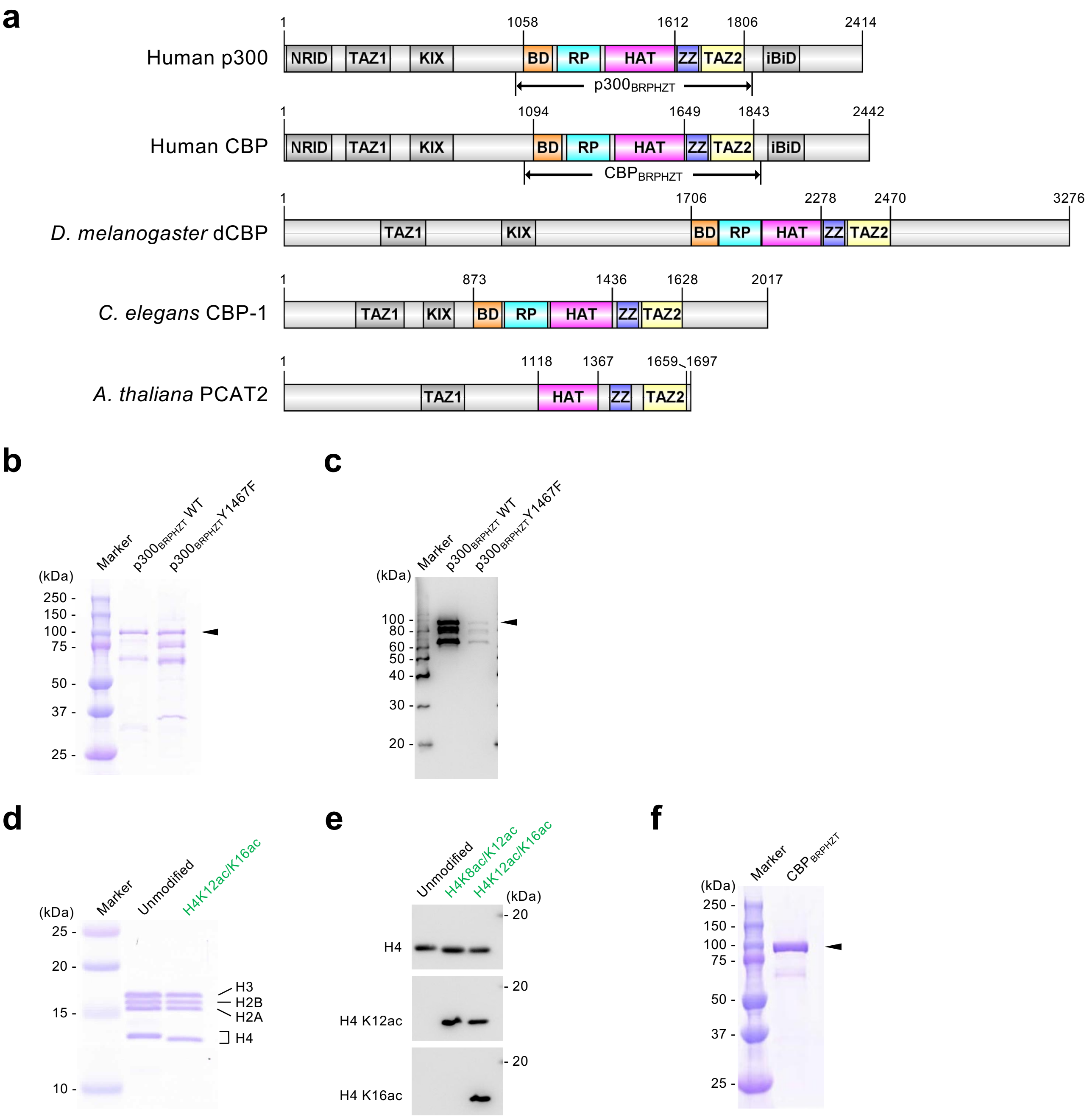

Supplementary Figure 1 Enzymes and nucleosome substrates used in this study. a Schematic representation of the domain architecture of metazoan p300/CREB-binding protein (CBP) homologs. NRID, nuclear receptor interaction domain; TAZ1, transcriptional adaptor zinc-finger domain 1; KIX, kinase-inducible domain of CREB-interacting domain; BD, bromodomain; RP, the RING and PHD zinc-fingers; HAT, histone acetyltransferase domain; ZZ, ZZ-type zinc-finger; TAZ2, transcriptional adaptor zinc-finger domain 2; and IBiD, IRF3-binding domain. The positions of the N- and C-termini and the start/end residues of the major domains are shown at the top of each scheme. The positions of the start/end residues of the human p300 and CBP constructs used in this study (i.e., p300$_{BRPHZT}$ and CBP$_{BRPHZT}$) are shown at the bottom of each scheme. **b** A Coomassie Brilliant Blue (CBB)-stained *sodium* dodecyl sulfate polyacrylamide gel electrophoresis (SDS-PAGE) image of the p300$_{BRPHZT}$ protein. The size of each band of molecular weight markers is indicated on the left. The position of p300$_{BRPHZT}$ is indicated by an arrowhead on the right. **c** Immunoblotting of autoacetylation of p300$_{BRPHZT}$. WT and Y1467F indicate wild-type and catalytically inactive p300 constructs, respectively. The position of p300$_{BRPHZT}$ is indicated by an arrowhead on the right. **d** Preparation of the nucleosome containing K12/K16-di-acetylated H4. A CBB-stained SDS-PAGE image of the reconstituted nucleosomes is shown. The positions of acetyllysine introduced into histone H4 in the nucleosome are given in green at the top. **e** Immunoblotting of residue-specific histone H4 acetylation of the reconstituted nucleosomes. The positions of acetyllysine introduced into histone H4 in the nucleosome are given in green at the top. The residue-specific histone acetylation recognition antibody used is shown on the left. Experiments in **c** and **e** were repeated independently three times with consistency. **f** A CBB-stained SDS-PAGE image of the CBP$_{BRPHZT}$ protein. The position of CBP$_{BRPHZT}$ is indicated by an arrowhead on the right. Experiments in **b**, **d**, and **f** were repeated independently twice with consistency.

Supplementary Figure 2

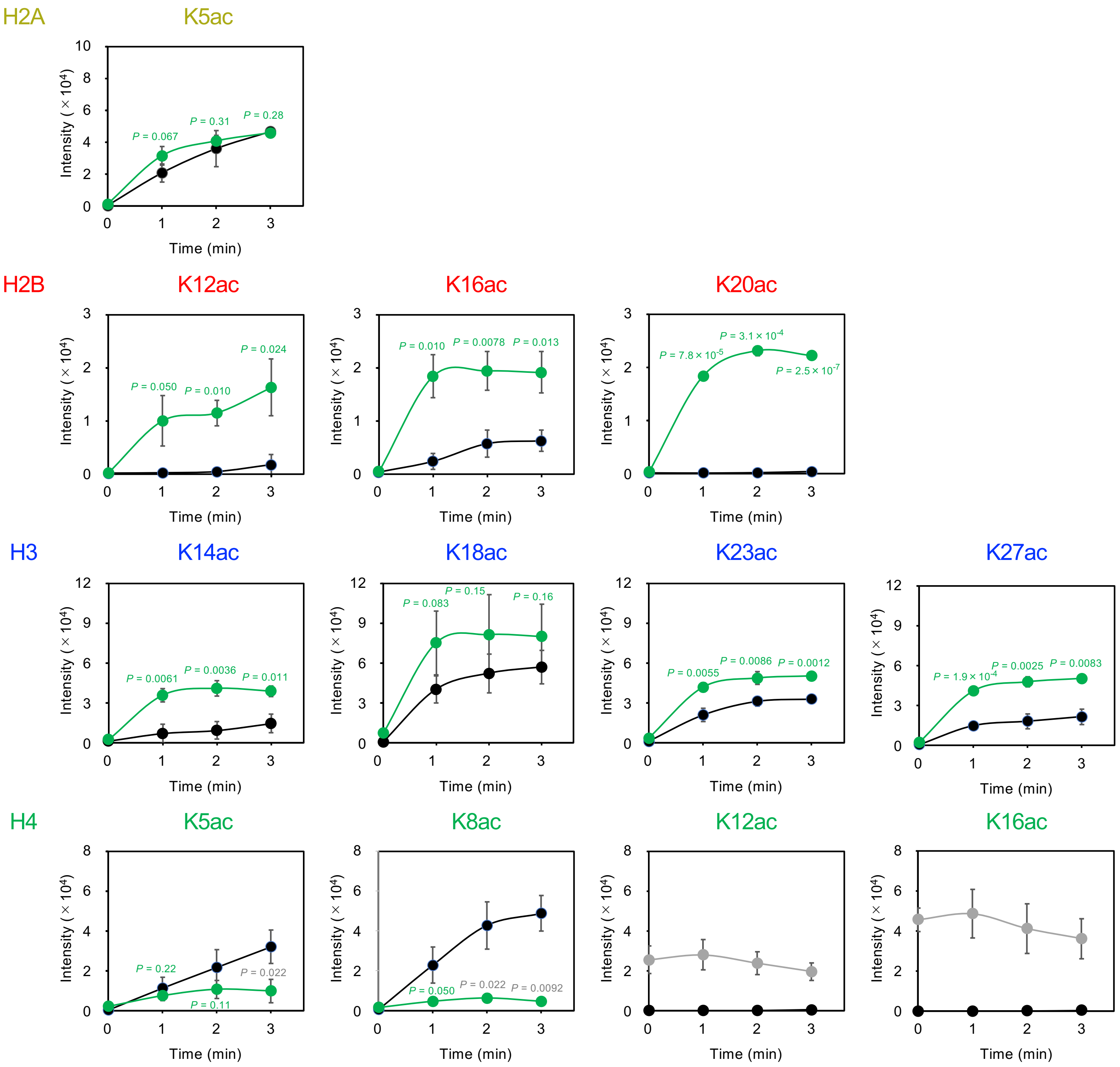

Supplementary Figure 2 In vitro acetyltransferase activity of p300$_{\mathrm{BRPHZT}}$ toward an H4-di-acetylated nucleosome. Residue-specific acetylation for each histone species detected by immunoblotting. The position of acetylation is shown above each panel. Black and green lines indicate the unmodified and the H4K12/K16-acetylated nucleosomes as substrates (1 μM), respectively. For pre-acetylated H4K12ac and H4K16ac residues, data with the H4K12/K16-acetylated nucleosome as substrate are shown as gray lines. The x-axis indicates the time course after the reaction in the presence of 1 μM p300$_{\mathrm{BRPHZT}}$ and 10 μM acetyl-CoA. The y-axis indicates the immunoblotting signal intensity. Data are mean ± SD from three independent experiments. *P*-value was calculated by a two-sample one-sided Welch's *t*-test for each time point. The alternative hypothesis is that the acetylated nucleosome is more acetylated by p300$_{\mathrm{BRPHZT}}$ than the unmodified nucleosome. *P*-value shown in gray is not a significant increase.

Supplementary Figure 3

H2A

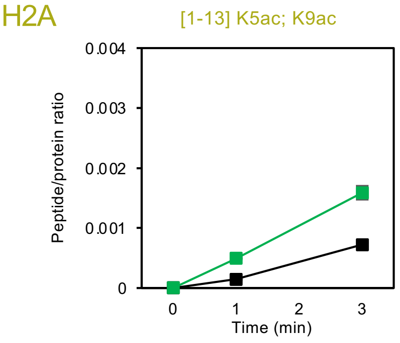

H2B

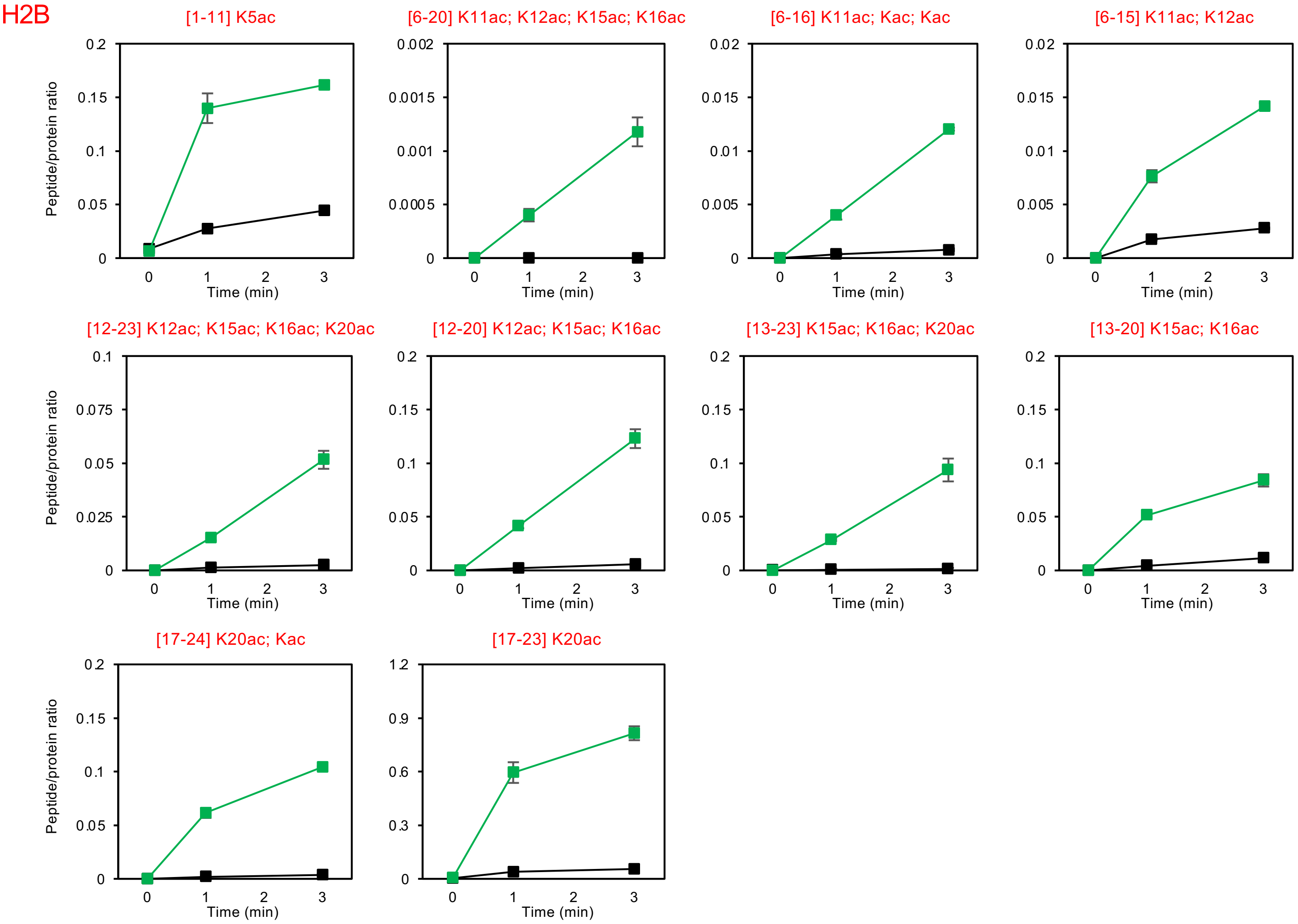

Supplementary Figure 3 (continued)

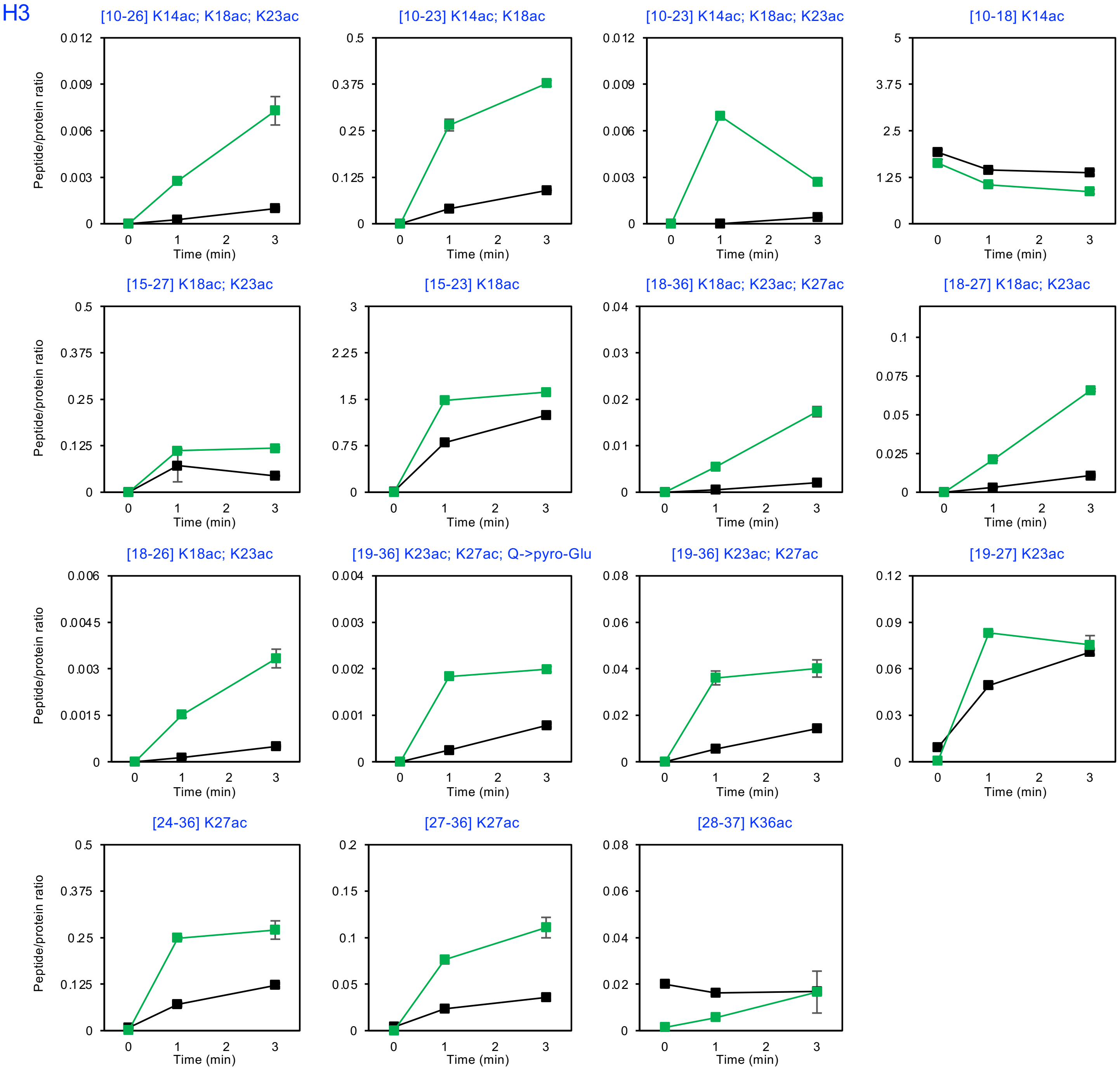

Supplementary Figure 3 (continued)

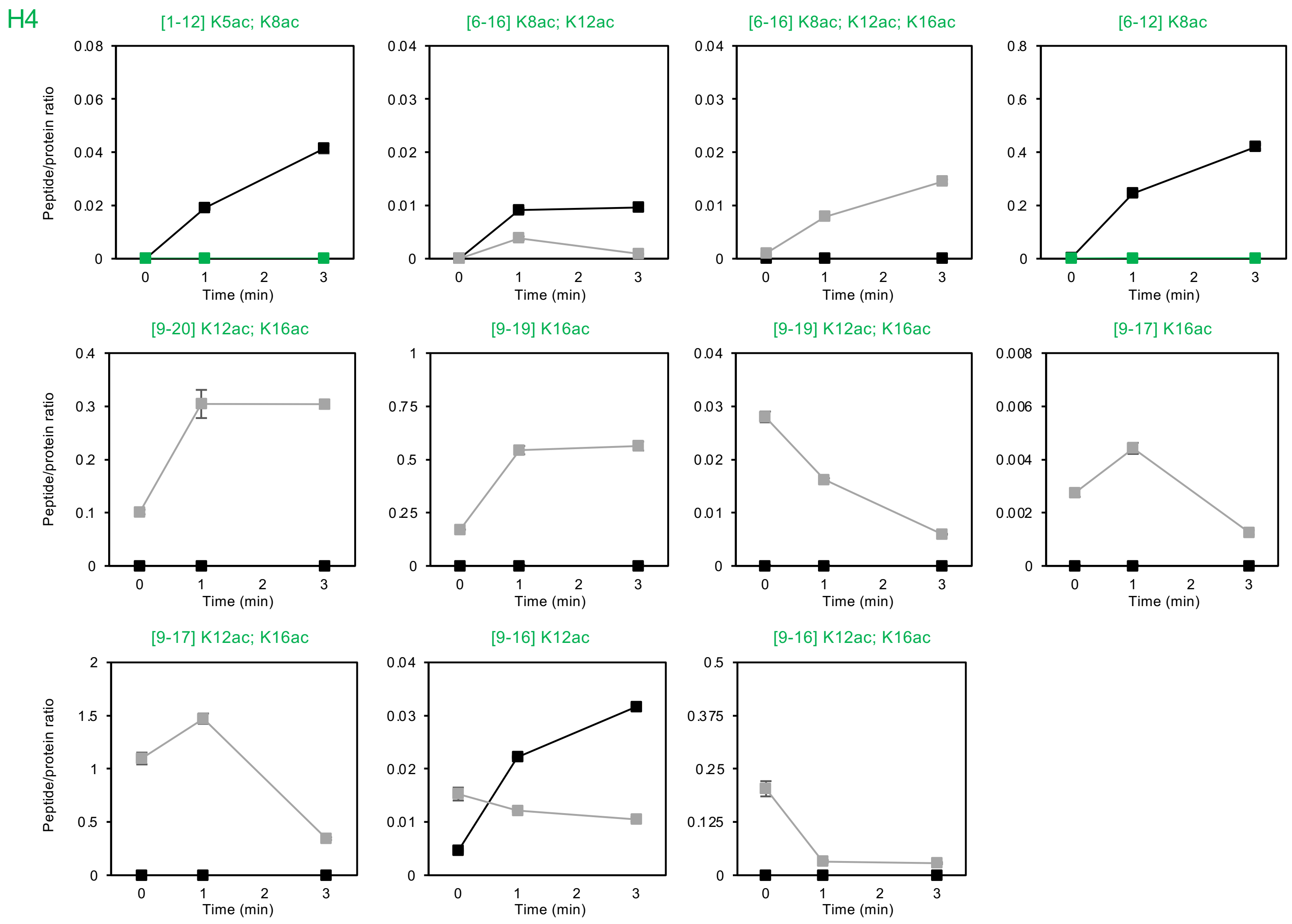

Supplementary Figure 3 Acetylated (ac) peptides of p300$_{BRPHZT}$-reacted histone N-terminal tails and their acetylation sites identified by mass spectrometry. The length of each histone peptide and the position of acetyllysine (Kac) are shown above each panel. Black and green lines indicate data with the unmodified and the H4K12/K16-acetylated nucleosomes as substrates (1 μM), respectively. Peptides containing H4K12ac or H4K16ac in the data with the H4K12/K16-acetylated nucleosome as substrate are shown as gray lines. The x-axis indicates the time course after the reaction in the presence of 1 μM p300$_{BRPHZT}$ and 10 μM acetyl-CoA. The y-axis indicates the ratio of the abundance of each peptide to the corresponding histone protein abundance. Data are mean ± standard error of the mean (SEM) from three technical replicates.

Supplementary Figure 4

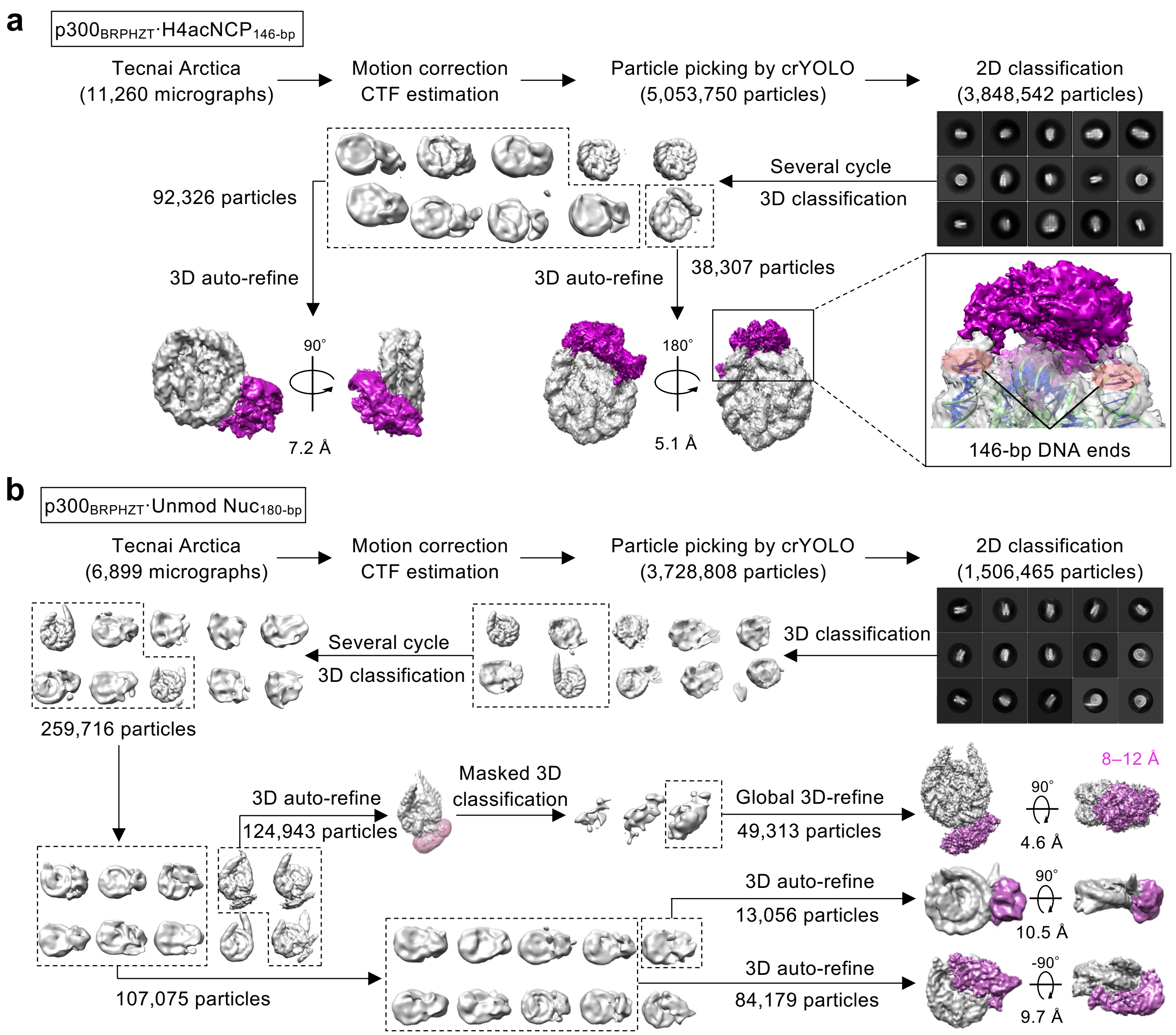

Supplementary Figure 4 Cryogenic electron microscopy (cryo-EM) workflow for proteins in complex with modified and unmodified nucleosomes. a The processing pipeline for p300$_{BRPHZT}$ in complex with H4K12/K16-acetylated nucleosome using 146-bp double-stranded DNA (H4acNCP$_{146\text{-bp}}$). **b** The cryo-EM processing pipeline for p300$_{BRPHZT}$ with unmodified nucleosome using 180-bp double-stranded DNA (Unmod Nuc$_{180\text{-bp}}$). For both pipelines, the resolution was estimated by the gold-standard Fourier shell correlation with the 0.143 criterion. Automated particle picking was performed with crYOLO v1.7.6. CTF, contrast transfer function.

Supplementary Figure 5

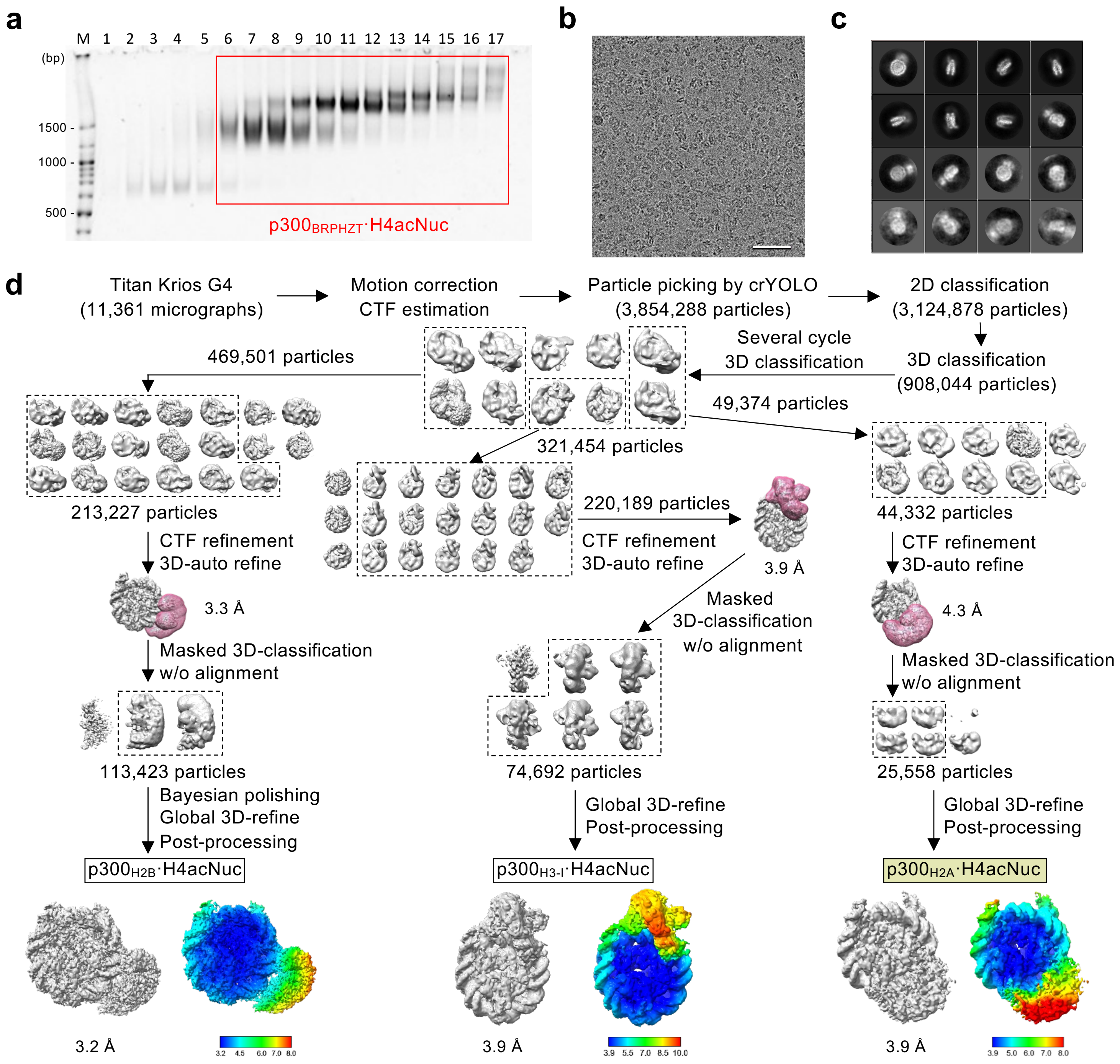

Supplementary Figure 5 (continued)

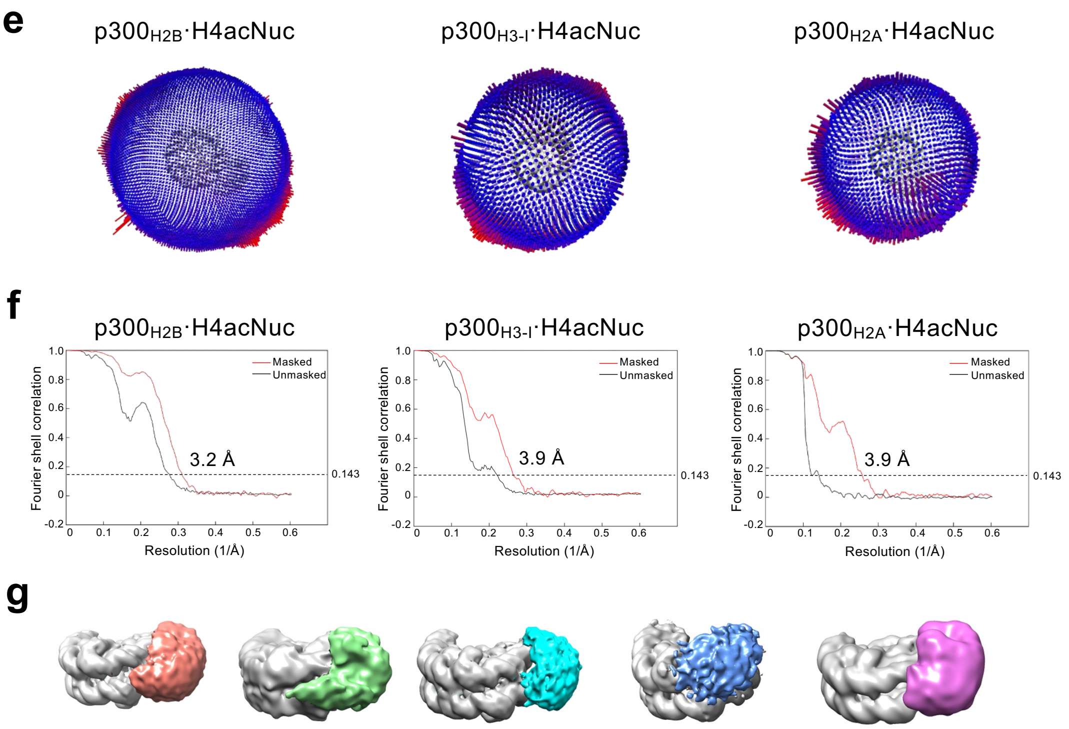

Supplementary Figure 5 Sample preparation and cryogenic electron microscopy (cryo-EM) structure determination of p300 protein in complex with reconstituted nucleosome (Titan Krios 300kV microscope). a $p300_{BRPHZT}$ and reconstituted nucleosome (H4acNuc) were incubated and isolated by glycerol-gradient (10–30%) with crosslinking. Fractions 1–17 were analyzed by electrophoretic mobility shift assay. Bands are labeled on the left. Fractions 6–17 in the red box were used for cryo-EM. **b** Example cryo-electron micrograph. Scale bar, 50 nm. Experiments in **a** and **b** were repeated independently twice with consistency. **c** Representative 2D class averages. **d** The processing pipeline for the $p300_{BRPHZT}$·H4acNuc complex. Local resolution (Å) was displayed on the sharpened full map. **e** Angular distribution of particle projections of the final reconstruction. **f** Gold-standard Fourier shell correlation curves of the final reconstitution. The resolution was estimated with the 0.143 criterion. Automated particle picking was performed with crYOLO v1.7.6. CTF, contrast transfer function. **g** Low-resolution complex structures similar to $p300_{H2B}$·H4acNuc in which H2BNT is located near the substrate-binding pocket of the histone acetyltransferase domain.

Supplementary Figure 6

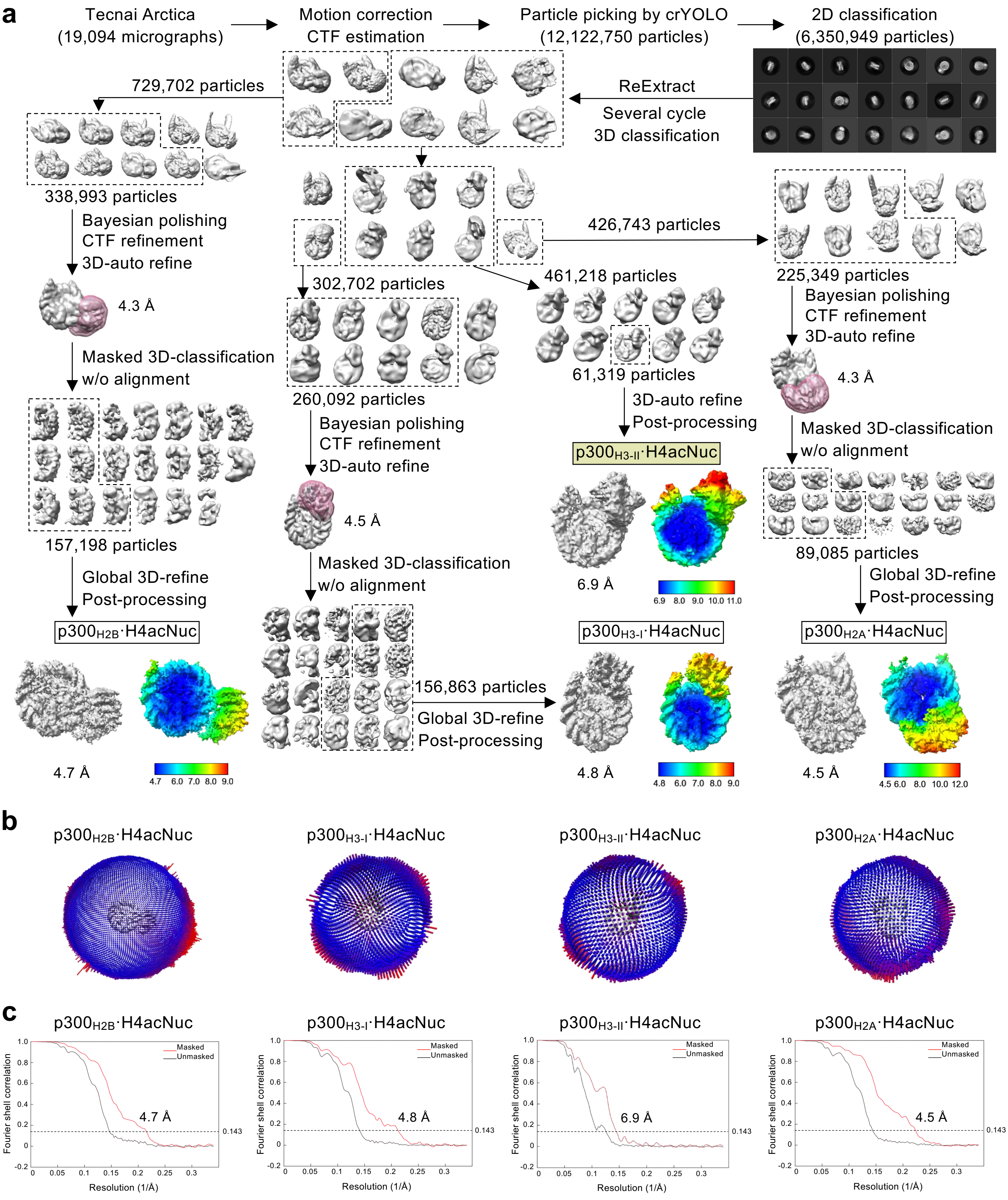

Supplementary Figure 6 Sample preparation and cryogenic electron microscopy structure determination of p300 protein in complex with reconstituted nucleosome (Tecnai Arctica 200kV microscope). a Processing pipeline for the $p300_{BRPHZT}$·reconstituted nucleosome (H4acNuc) complex. Local resolution (Å) was displayed on the sharpened full map. **b** Angular distribution of particle projections of the final reconstruction. **c** Gold-standard Fourier shell correlation curves of final reconstitution. The resolution was estimated with the 0.143 criterion. Automated particle picking was performed with crYOLO v1.7.6. CTF, contrast transfer function.

Supplementary Figure 7

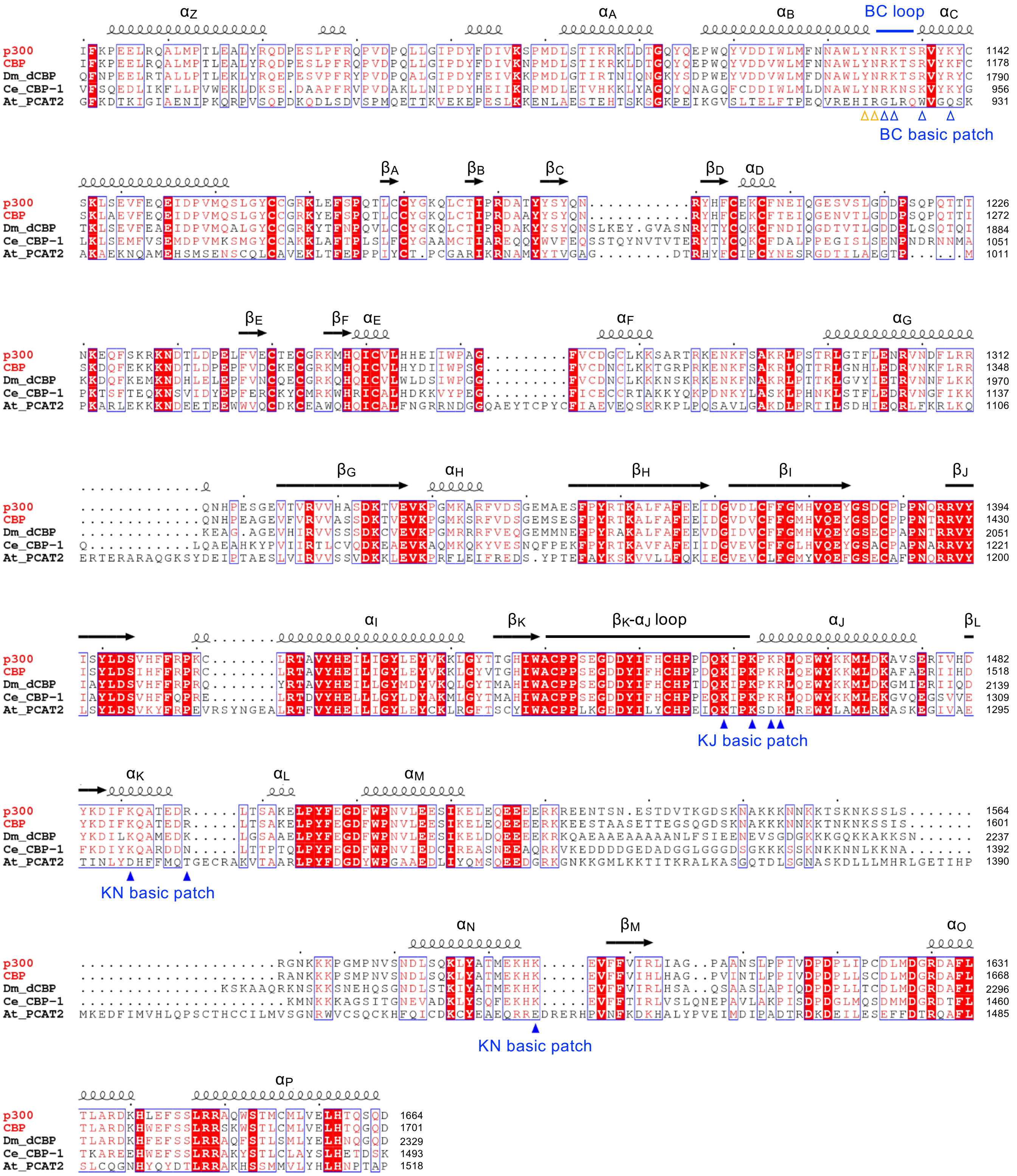

Supplementary Figure 7 Sequence alignment of BRPH in p300/CREB-binding protein (CBP) homologs. Homology between human p300 and CBP, *D. melanogaster* (Dm) dCBP, *C. elegans* (Ce) CBP-1, and *A. thaliana* (At) p300/CBP AcetylTransferase-related protein 2 (PCAT2) is shown. Residue numbers on the C-terminal side of each row are shown on the right. Conserved or similar residues are shown in red and surrounded by blue boxes. The completely conserved residues are shown in white letters on a red background. The positions and numbers of α-helices and β-strands are indicated at the top of the alignment. The α-helices are numbered to match the bromodomain helix numbers (αz to αC). K/R residues comprising the two DNA-interactive basic patches in human p300 are indicated by filled blue arrowheads at the bottom (KJ basic patch: K1456, K1459, K1461, and R1462; KN basic patch: K1488, R1494, and K1592). Residues involved in the recognition of acetyllysine inside the bromodomain pocket (Y1131 and N1132) are indicated by open orange arrowheads. K/R residues comprising the third basic patch around the BC loop of the bromodomain (BC basic patch; R1133, K1134, R1137, and K1140) are indicated by open blue arrowheads.

Supplementary Figure 8

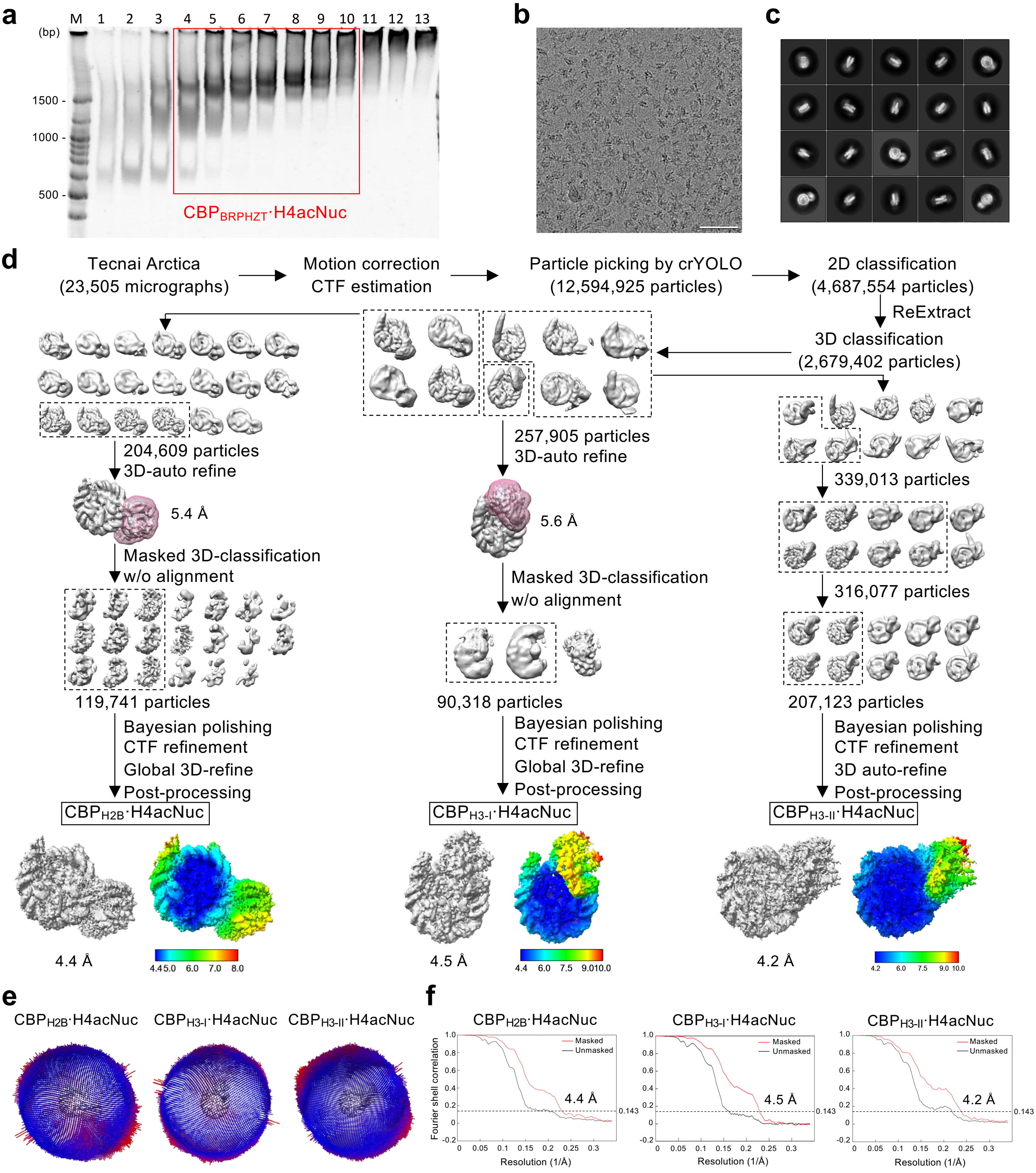

Supplementary Figure 8 Sample preparation and cryogenic electron microscopy (cryo-EM) structure determination of CREB-binding protein (CBP) in complex with reconstituted nucleosome. a CBP_{BRPHZT} and the reconstituted nucleosome (H4acNuc) were incubated and isolated by glycerol-gradient (10–40%) with crosslinking. Fractions 1–13 were analyzed by electrophoretic mobility shift assay. Bands are labeled on the left. Fractions 4–10 (red box) were used for cryo-EM. **b** Example cryo-electron micrograph. Scale bar, 50 nm. Experiments in **a** and **b** were repeated independently twice with consistency. **c** Representative 2D class averages. **d** The microscopy processing pipeline for CBP_{BRPHZT}·H4acNuc complex. Local resolution (Å) is displayed on the sharpened full map. **e** Angular distribution of particle projections of the final reconstruction. **f** Gold-standard Fourier shell correlation curves of the final reconstitution. The resolution was estimated with the 0.143 criterion. Automated particle picking was performed with crYOLO v1.7.6. CTF, contrast transfer function.

Supplementary Figure 9

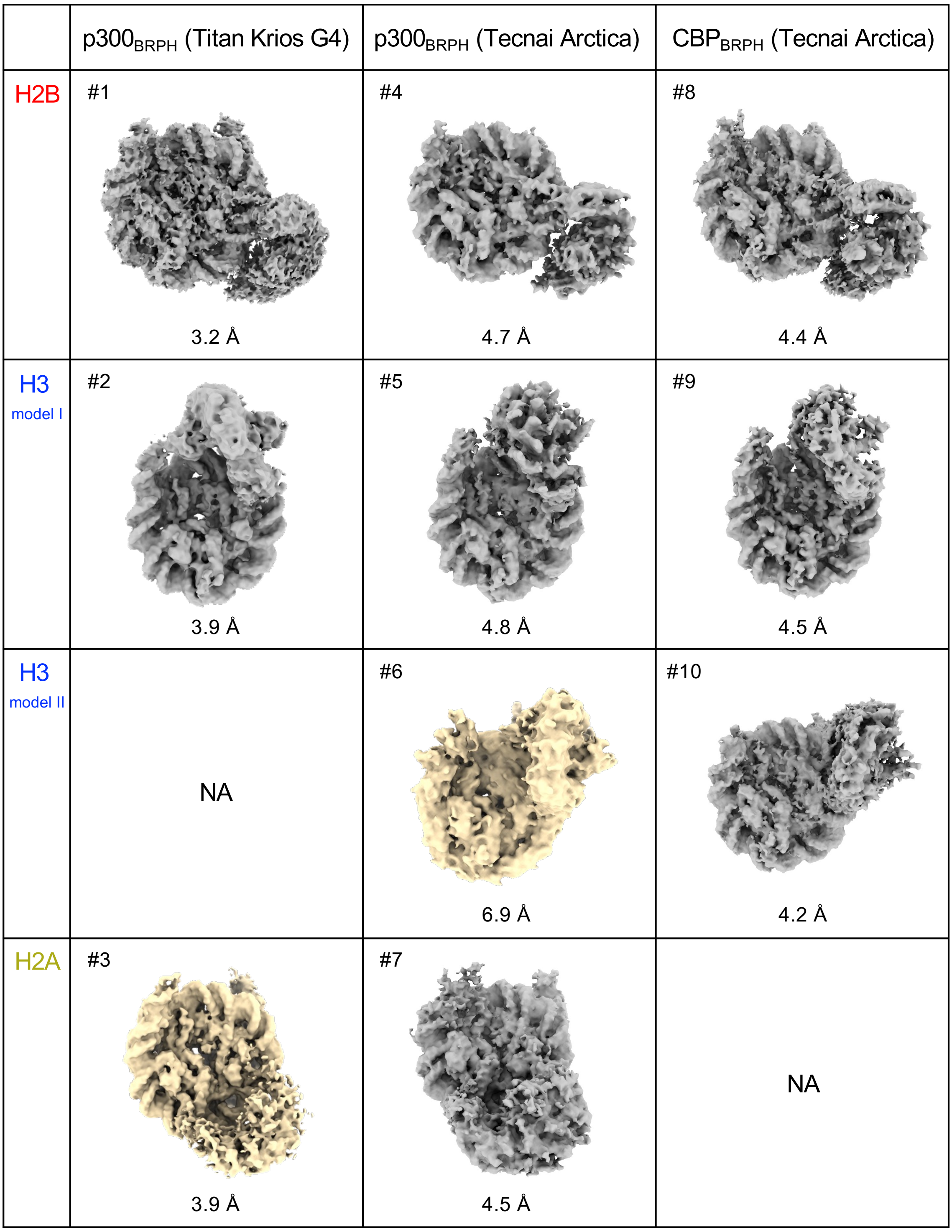

Supplementary Figure 9 All cryogenic electron microscopy (cryo-EM) maps of $p300_{BRPH}$/CREB-binding protein $(CBP)_{BRPH}$ complexed with H4acNuc. The resolution was estimated by the gold-standard Fourier shell correlation with the 0.143 criterion. Cryo-EM maps for which the atomic coordinates were determined are shown in dark gray. Those for which the atomic coordinates could not be determined are shown in light yellow. NA, not available.

Supplementary Figure 10

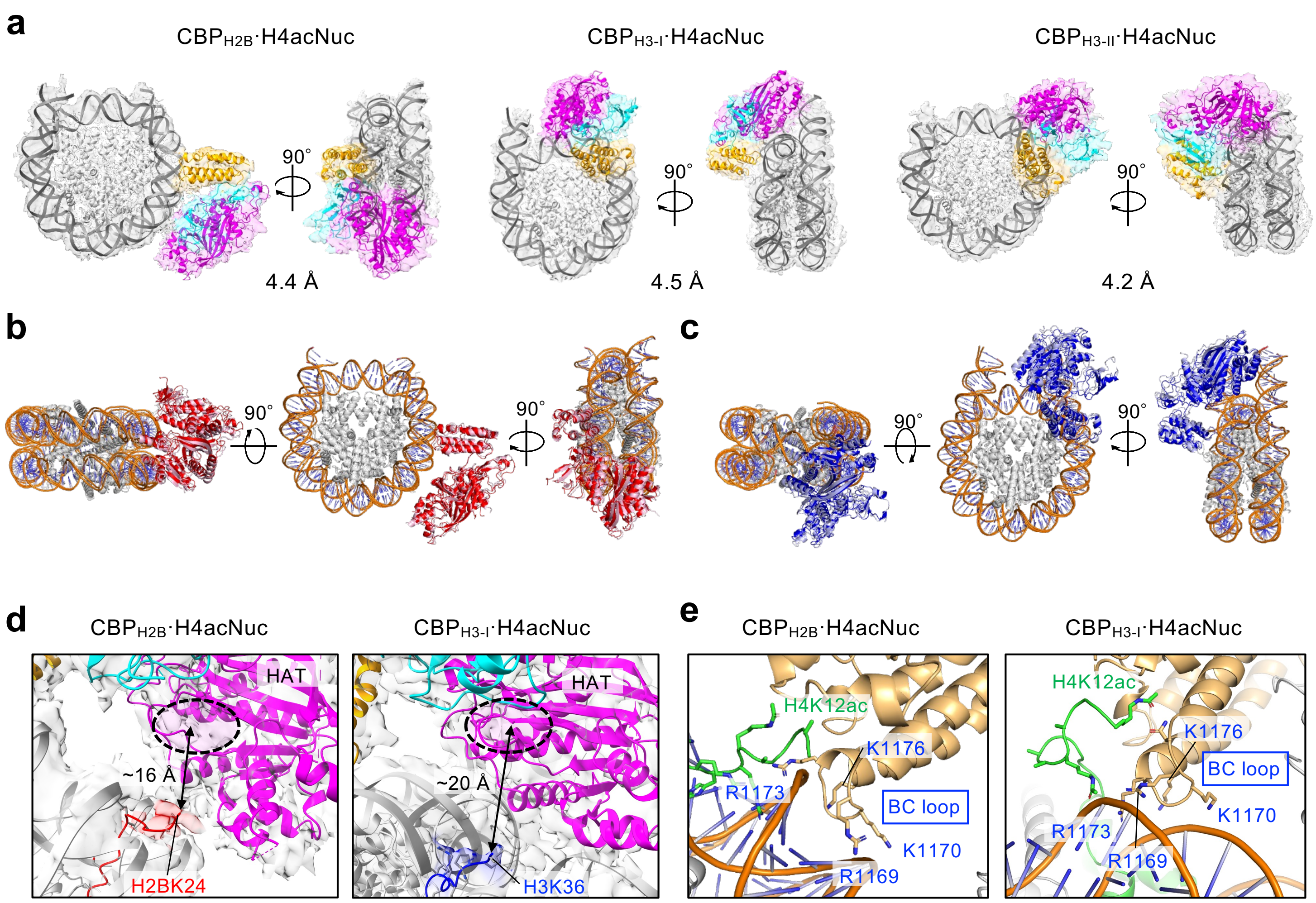

Supplementary Figure 10 Cryogenic electron microscopy (cryo-EM) structure of CREB-binding protein (CBP) in complex with reconstituted nucleosome. a Various conformations of CBP_{BRPH} and reconstituted nucleosome (H4acNuc) in cryo-EM maps and structural model. Left, CBP_{H2B}·H4acNuc (#8 in Supplementary Fig. 9); center, $CBP_{H3\text{-}I}$·H4acNuc (#9); right, $CBP_{H3\text{-}II}$·H4acNuc (#10). **b** Superimposition of CBP_{H2B}·H4acNuc (red) on $p300_{H2B}$·H4acNuc (pink). **c** Superimposition of $CBP_{H3\text{-}I}$·H4acNuc (blue) on $p300_{H3\text{-}I}$·H4acNuc (light blue). **d** Close-up views (#8 and #9) of the structural model and cryo-EM maps of H4K12acK16ac binding by BD of each complex structure. Color code: orange, CBP BD; cyan, CBP RP; magenta, CBP HAT; green, K12/K16-acetylated H4. **e** Close-up views (#8 and #9) of the interaction region by lysine and arginine in the BC loop of the CBP of each complex structure.

Supplementary Figure 11

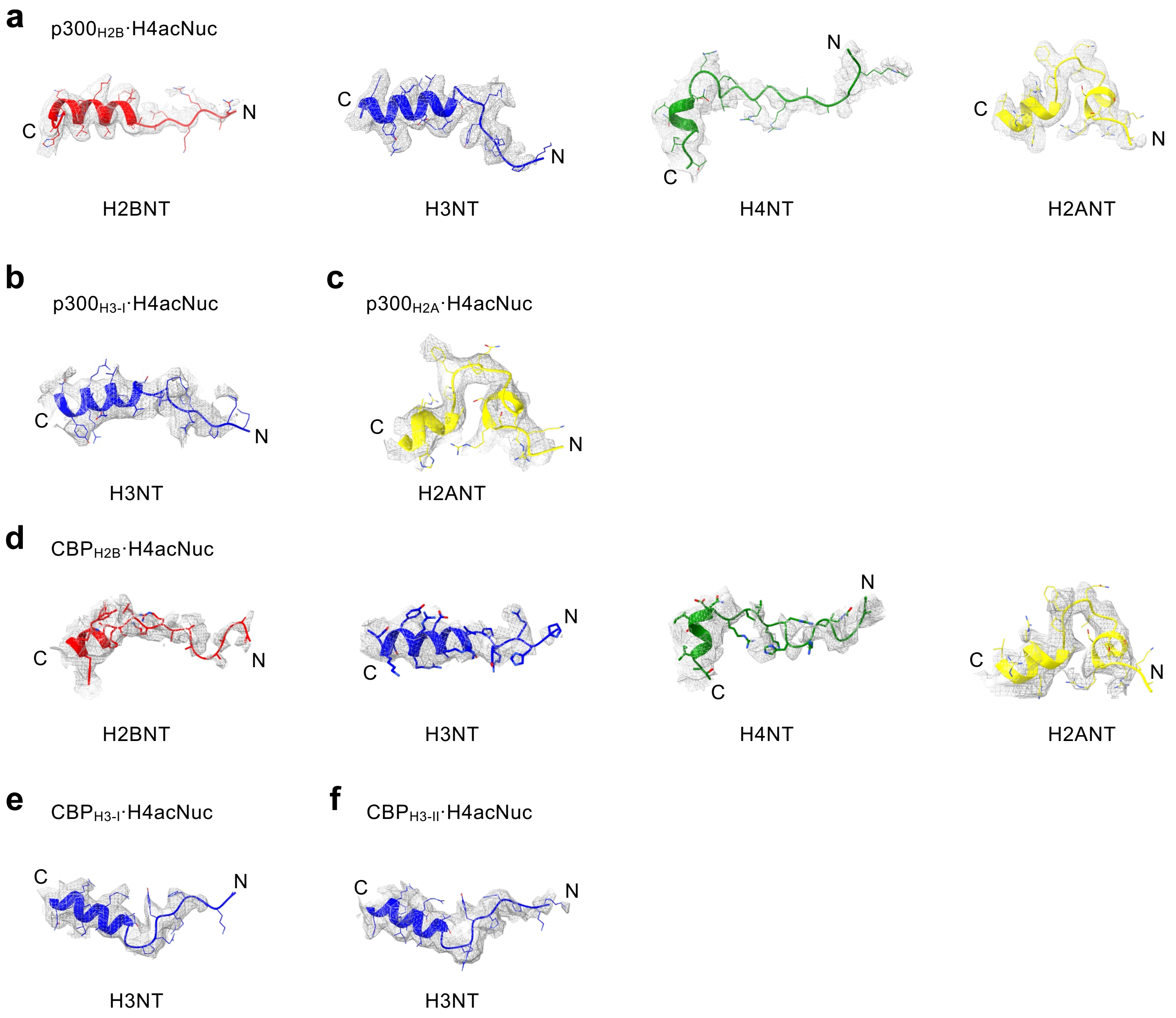

Supplementary Figure 11 Cryogenic electron microscopy maps of histone N-terminal tails (NTs) in close proximity to histone acetyltransferase domain of p300$_{\mathrm{BRPH}}$/CREB-binding protein (CBP)$_{\mathrm{BRPH}}$. a Histone NTs of p300$_{\mathrm{H2B}}$ in complex with reconstituted nucleosome (H4acNuc). **b** H3NT of p300$_{\mathrm{H3\text{-}I}}$·H4acNuc. **c** H2ANT of p300$_{\mathrm{H2A}}$·H4acNuc. **d** Histone NTs of CBP$_{\mathrm{H2B}}$·H4acNuc. **e** H3NT of CBP$_{\mathrm{H3\text{-}I}}$·H4acNuc. **f** H3NT of CBP$_{\mathrm{H3\text{-}II}}$·H4acNuc. Each density and the corresponding structural model are depicted in the gray mesh and colored ribbon diagrams, respectively. N and C indicate the N- and C-terminal sides of the histone tail, respectively.

Supplementary Figure 12

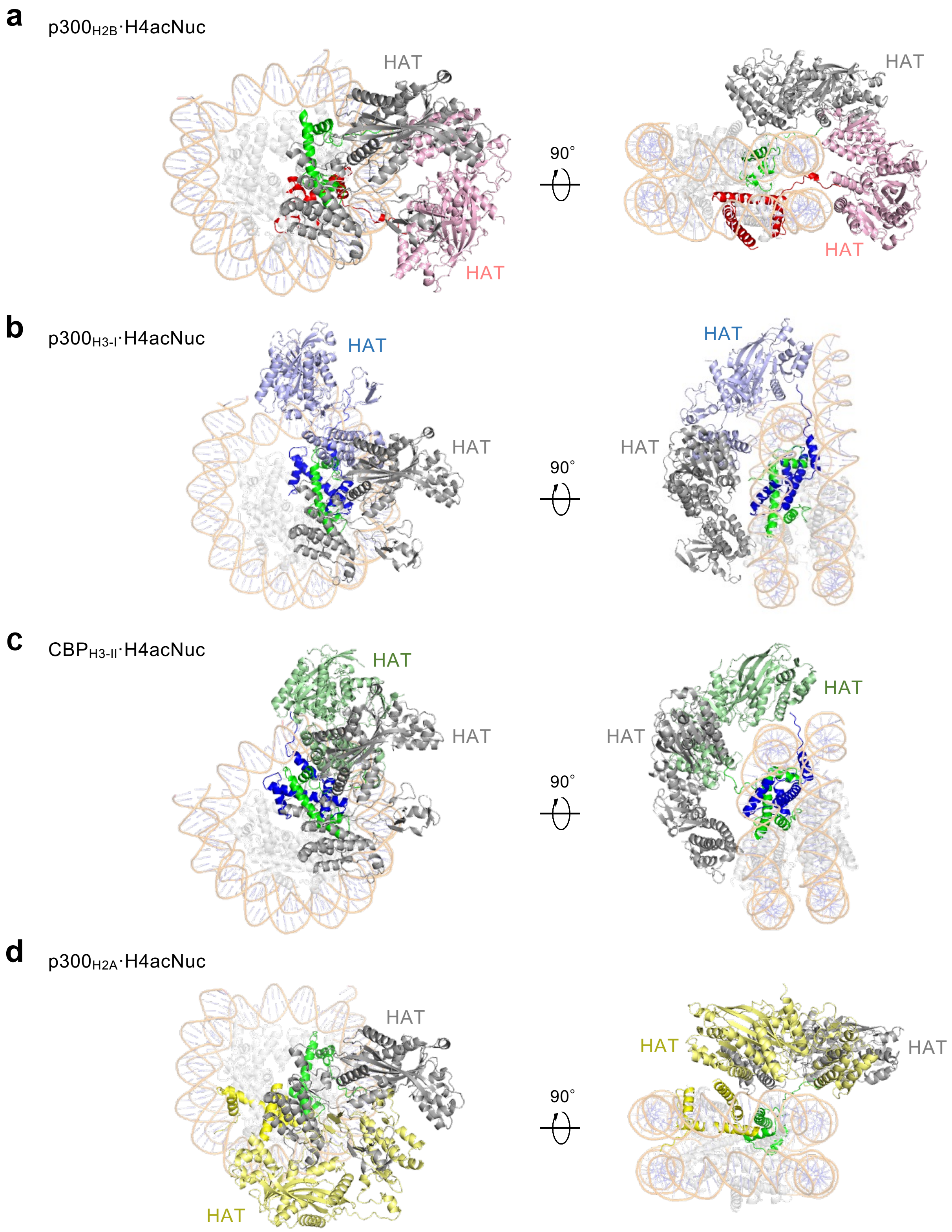

Supplementary Figure 12 Structural comparison with the known p300·nucleosome complex (PDB ID: 7W9V). a Superimposition with $p300_{H2B}$ in complex with H4acNuc. **b** Superimposition with $p300_{H3\text{-}I}$·H4acNuc. **c** Superimposition with CREB-binding protein $(CBP)_{H3\text{-}II}$·H4acNuc. **d** Superimposition with $p300_{H2A}$·H4acNuc. The structures are superimposed with the position of the nucleosome aligned. The catalytically inactive p300($BRPH_{\Delta AIL}Z$) protein of 7W9V is shown in gray. $p300_{BRPH}$ or CBP_{BRPH} in $p300_{H2B}$·H4acNuc, $p300_{H3\text{-}I}$·H4acNuc, $CBP_{H3\text{-}II}$·H4acNuc, and $p300_{H2A}$·H4acNuc are shown in light red, light blue, light green, and light yellow, respectively. The position of each histone acetyltransferase (HAT) is indicated by the same color code. Histones in close proximity to each HAT are highlighted with the following coloring: **a** H2B (red) and H4 (green), **b** and **c** H3 (blue) and H4 (green), **d** H2A (yellow) and H4 (green).

Supplementary Figure 13

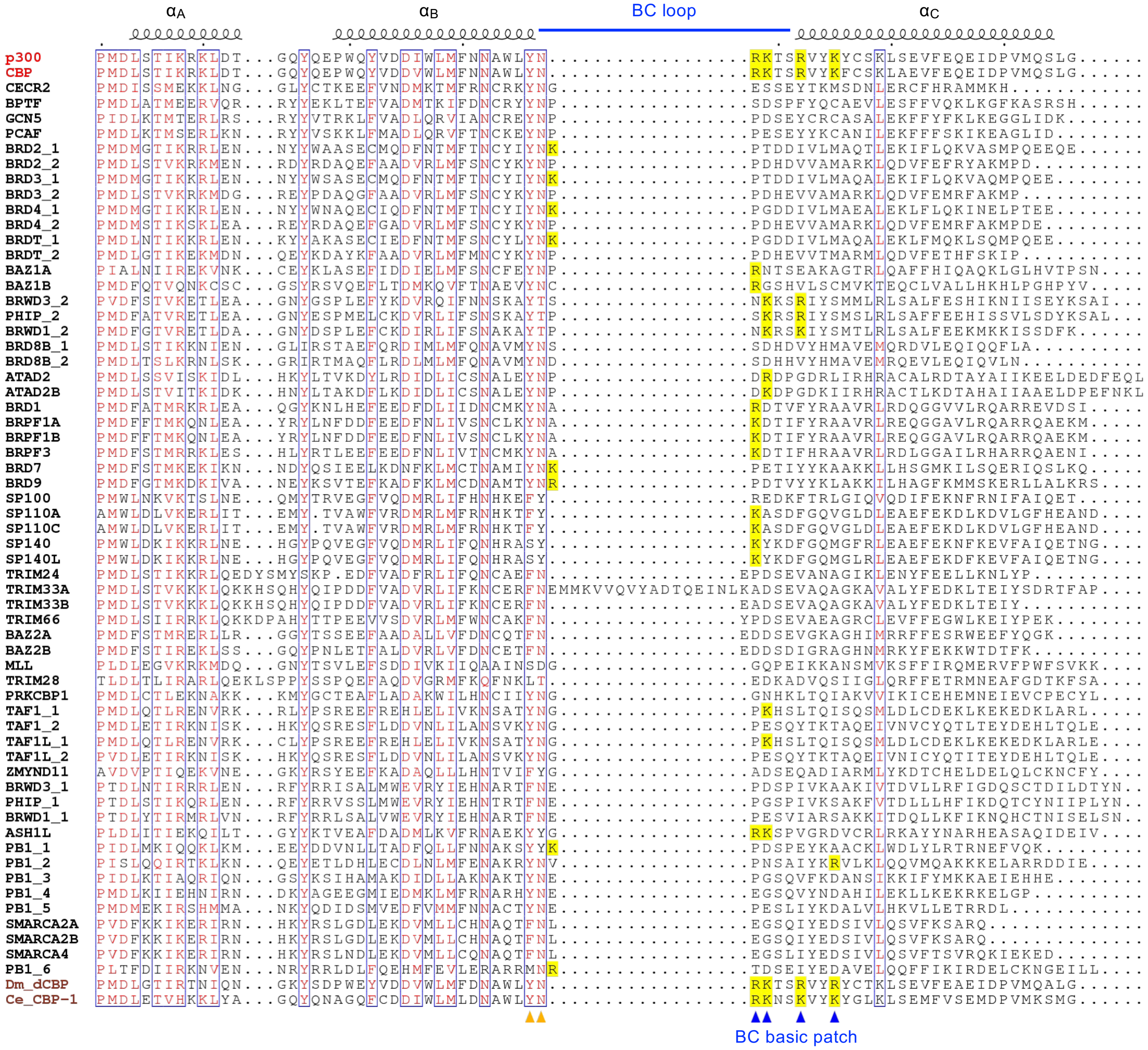

Supplementary Figure 13 Sequence alignment around the BC loop of the bromodomains. The amino acid sequences of all 61 human bromodomains and the *D. melanogaster* and *C. elegans* p300/CBP homologs (i.e., Dm_dCBP and Ce_CBP-1 shown in brown) are aligned. The positions of the BC loop and three α-helices composing the bromodomain are shown on the top in black and blue, respectively. Protein names of human bromodomains other than p300 and CBP are shown in black on the left. Conserved or similar residues are shown in red and surrounded by blue boxes. Residues involved in the recognition of acetyllysine inside the bromodomain pocket (Y1131 and N1132) are indicated by filled orange arrowheads at the bottom. K/R residues comprising the basic patch around the BC loop of the bromodomain (BC basic patch; R1133, K1134, R1137, and K1140 of human p300) are indicated by a yellow background and filled blue arrowheads at the bottom.

Supplementary Figure 14

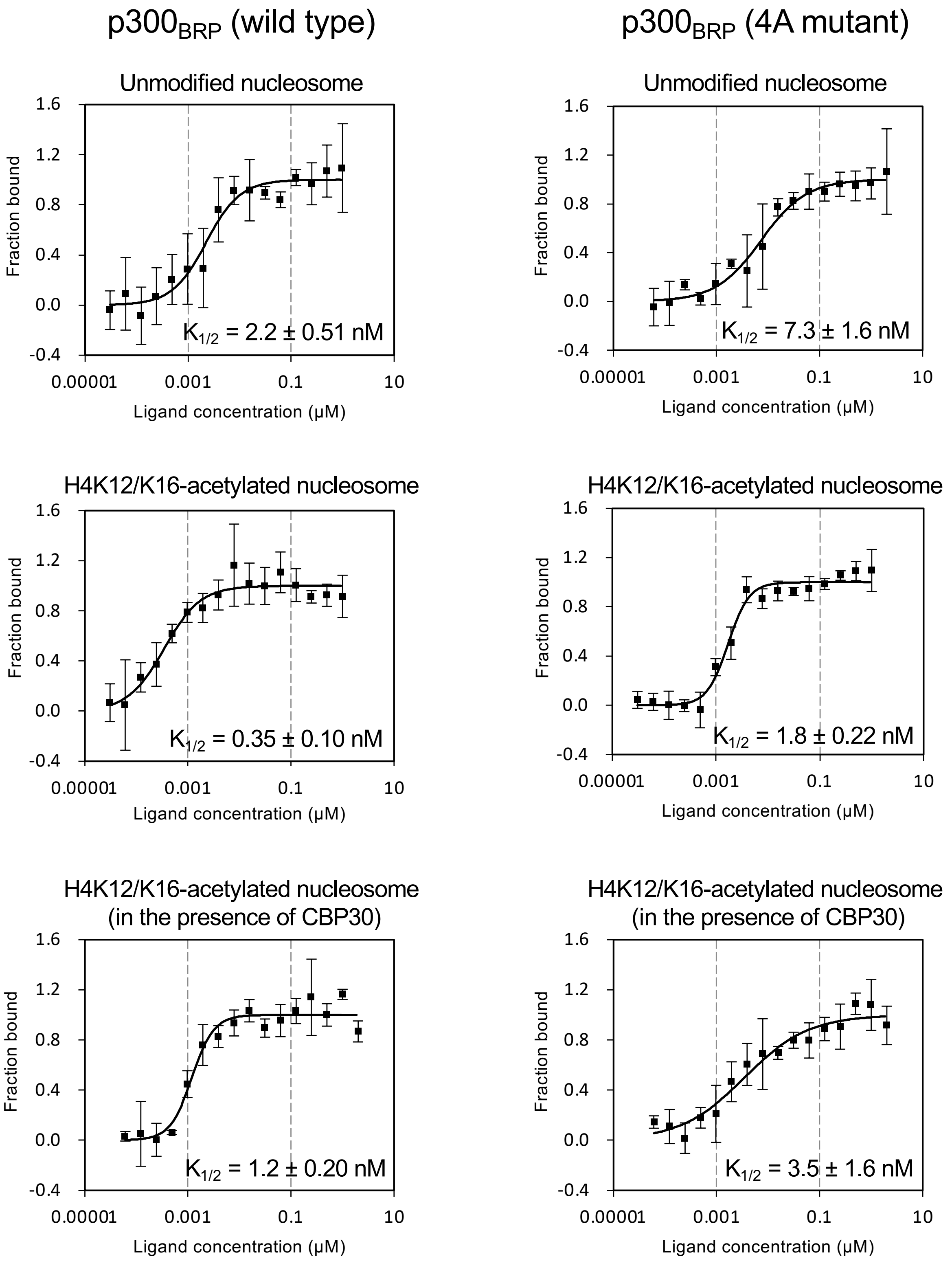

Supplementary Figure 14 Binding analysis between p300 bromodomain and nucleosomes measured by microscale thermophoresis. Binding analysis was performed for wild-type (left) or 4A-substituted mutant (right) $p300_{BRP}$ (residues 1048–1282; 4A, mutations of R1133A, K1134A, R1137A, and K1140A) to the nucleosomes shown above each panel. $K_{1/2}$ values are shown within each panel. Data are mean ± SEM from three independent experiments. CBP30 is an inhibitor that prevents the bromodomain pocket of p300 from binding to the acetylated histone N-terminal tails.

Supplementary Figure 15

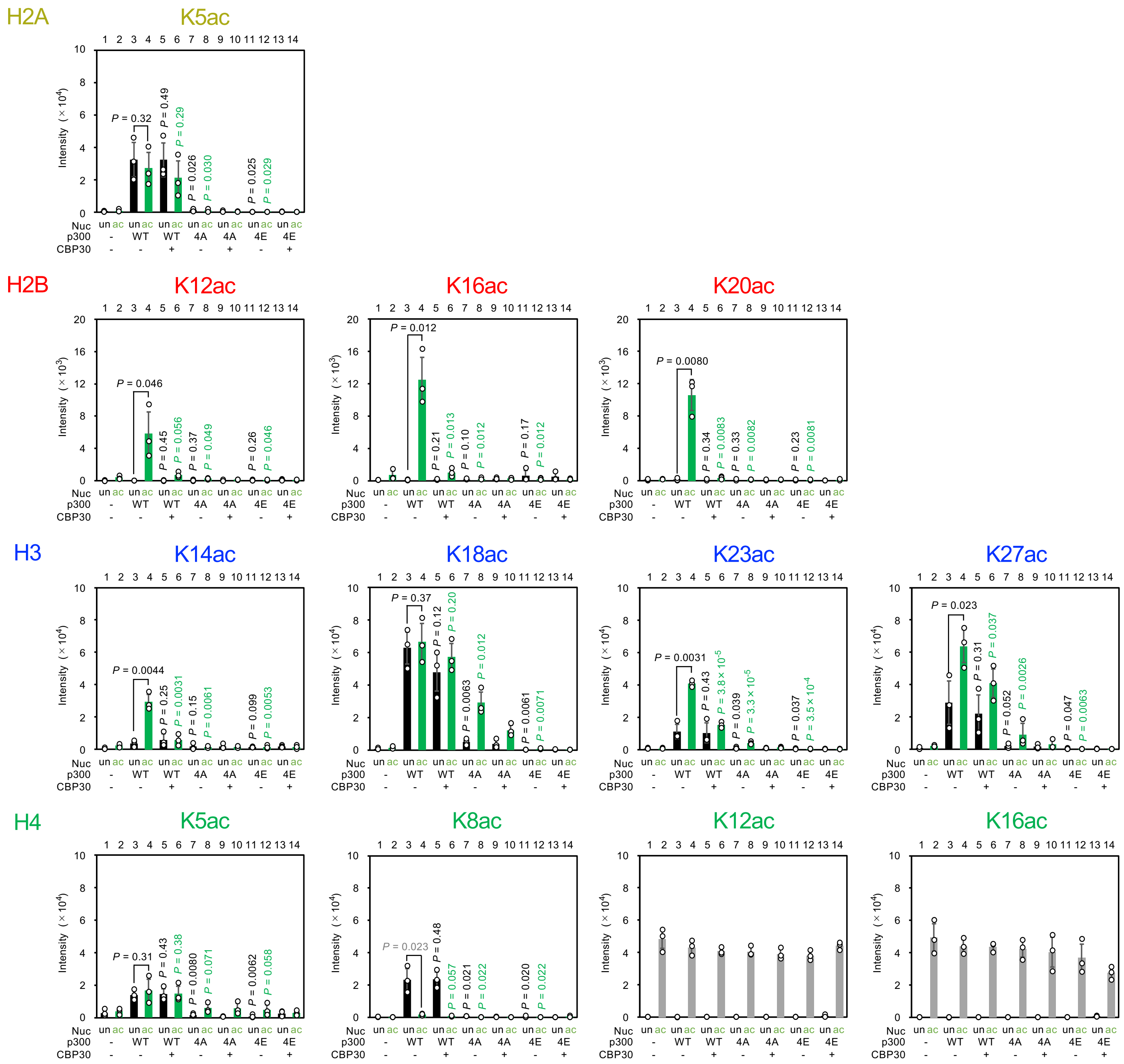

Supplementary Figure 15 Effect of p300 mutations on its acetyltransferase activity toward the H4-di-acetylated nucleosome. Residue-specific histone acetylation detected by immunoblotting for each histone species. The position of acetylation is shown above each panel. Nucleosome (Nuc): un (black), unmodified; ac (green), H4K12/K16-acetylated. p300: WT, wild-type $p300_{BRPHZT}$; 4A, $p300_{BRPHZT}$ with mutations of R1133A, K1134A, R1137A, and K1140A; 4E, $p300_{BRPHZT}$ with mutations of R1133E, K1134E, R1137E, and K1140E; and CBP30: -, none; +, 10 µM. CBP30 is an inhibitor that prevents the bromodomain pocket of p300 from binding to the acetylated histone N-terminal tails. The y-axis indicates the immunoblotting signal intensity at 1 min after the reaction. Data are mean ± SD from three independent experiments. For pre-acetylated H4K12ac and H4K16ac residues, data with the H4K12/K16-acetylated nucleosome as substrate are shown as gray bars. *P*-value was calculated by a two-sample one-sided Welch's *t*-test. The alternative hypothesis is as follows: lane 4, increase vs. lane 3; lanes 5, 7, and 11, decrease vs. lane 3; lanes 6, 8, and 12, decrease vs. lane 4. *P*-value shown in gray is not a significant increase.

Supplementary Figure 16

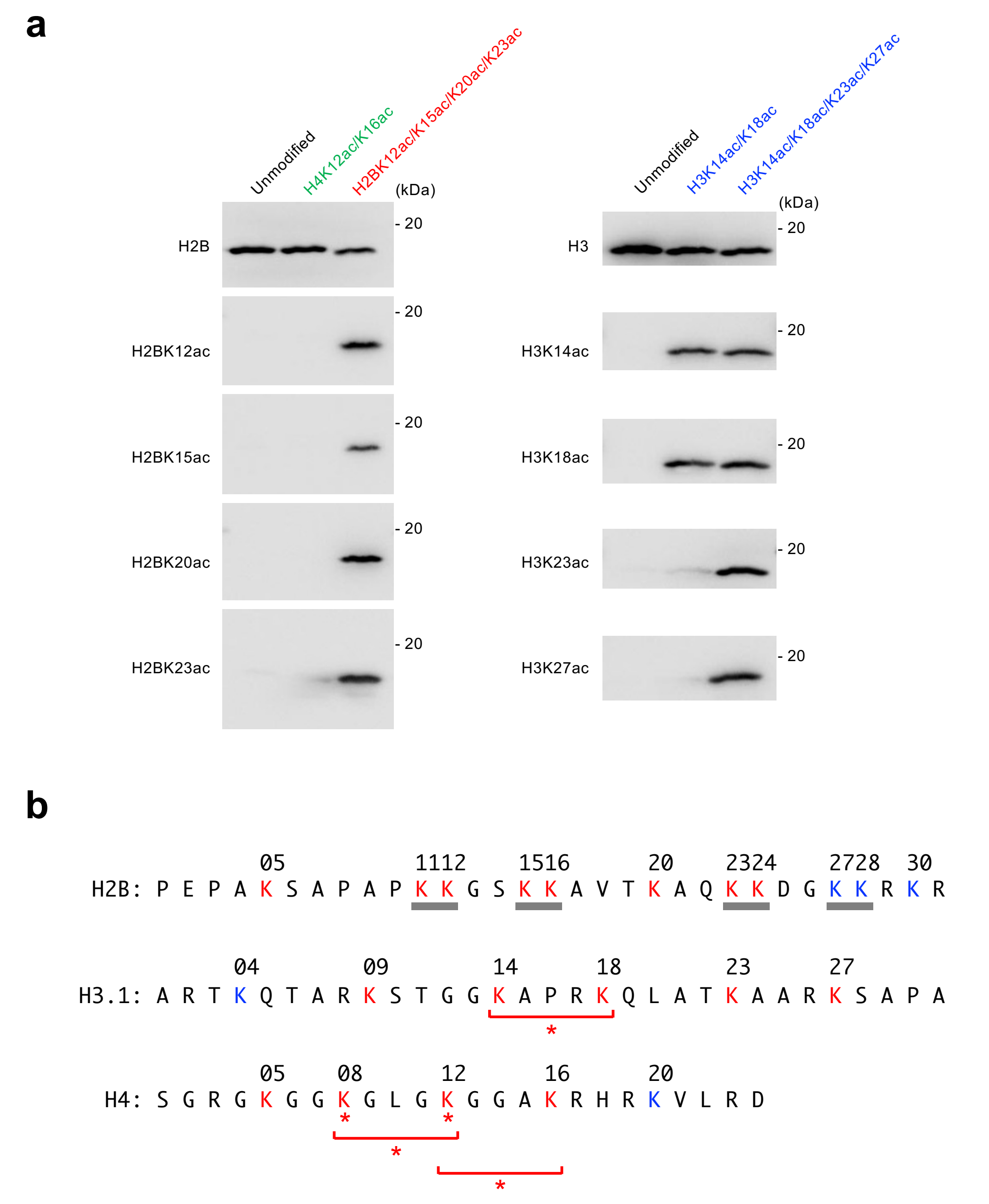

Supplementary Figure 16 Lysine acetylation in the histone N-terminal tails (NTs). a Immunoblotting of residue-specific histone acetylation of the reconstituted nucleosomes. Left, H4-di-acetylated or H2B-tetra-acetylated nucleosome; right, H3-di-acetylated or tetra-acetylated nucleosomes. The position(s) of acetyllysine introduced into histone H4, H2B, or H3 in the nucleosome are shown above each image. The residue-specific histone acetylation recognition antibody used is shown to the left of each image. Immunoblotting experiments were repeated independently three times with consistency. **b** Amino acid sequence of human histone NTs. Histone names are shown on the left. The type of H2B is 1-J. Lysine (K) residues that p300/CBP reportedly acetylates[19] are indicated in red, and those it does not acetylate in blue. Sequences with contiguous lysine residues (KK) are indicated by gray bars at the bottom. Kac residues or combinations of Kac residues to which p300 bromodomain binds are indicated by asterisks and underlines, respectively. A binding threshold was applied at a K_D of ~100 μM[15].

Supplementary Figure 17

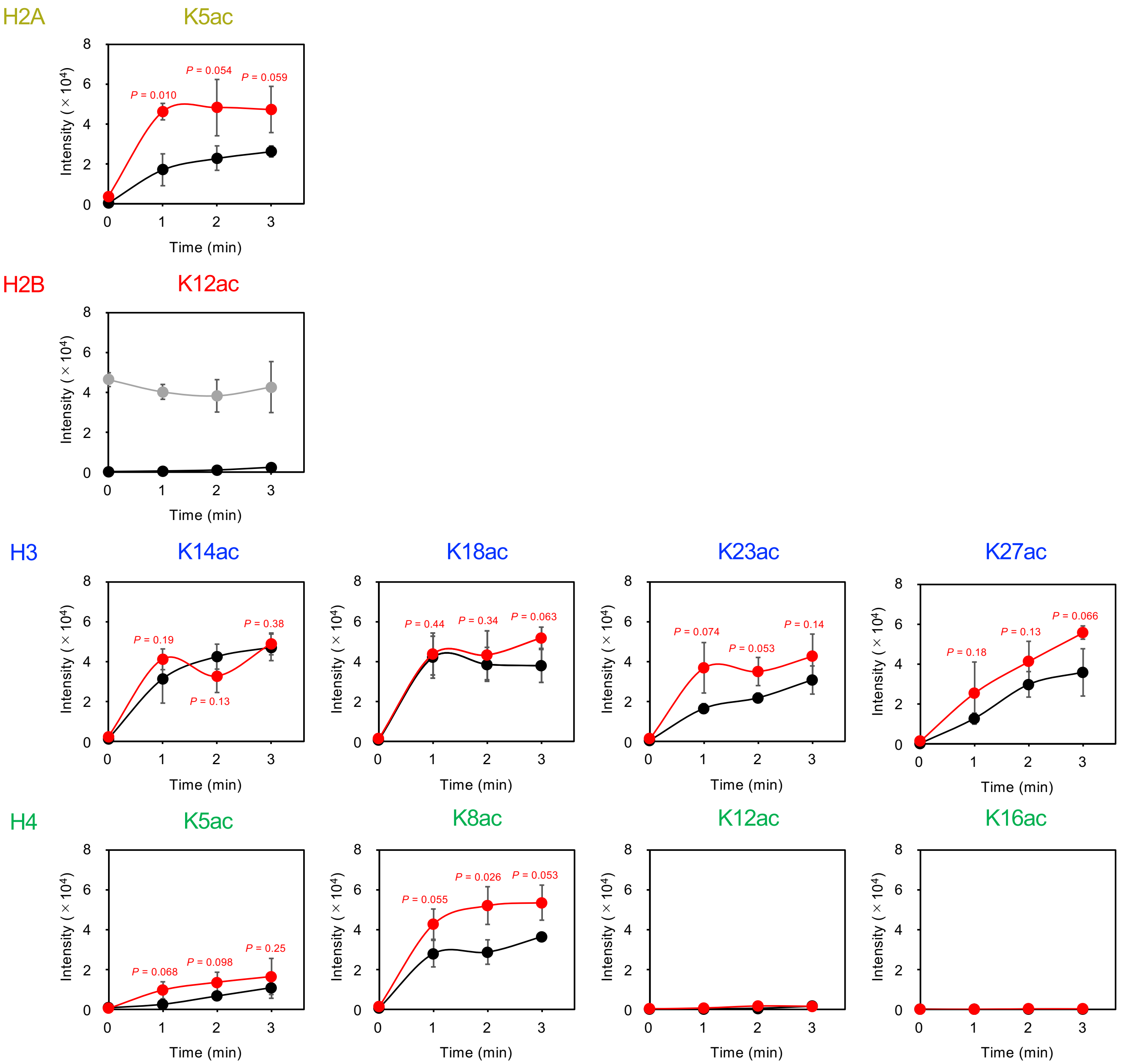

Supplementary Figure 17 In vitro acetyltransferase activity of p300$_{BRPHZT}$ toward an H2B-tetra-acetylated nucleosome. Residue-specific histone acetylation detected by immunoblotting for each histone species. The position of acetylation is shown above each panel. Black and red lines indicate the unmodified- and the H2BK12/K15/K20/K23-acetylated nucleosomes as substrates (1 µM), respectively. For pre-acetylated H2BK12ac residue, data with the H2BK12/K15/K20/K23-acetylated nucleosome as substrate is shown as gray line. The x-axis indicates the time course after the reaction in the presence of 1 µM p300$_{BRPHZT}$ and 10 µM acetyl-CoA. The y-axis indicates the immunoblotting signal intensity. Data are mean ± SD from three independent experiments. *P*-value was calculated by a two-sample one-sided Welch's *t*-test for each time point. The alternative hypothesis is that the acetylated nucleosome is more acetylated by p300$_{BRPHZT}$ than the unmodified nucleosome.

Supplementary Figure 18

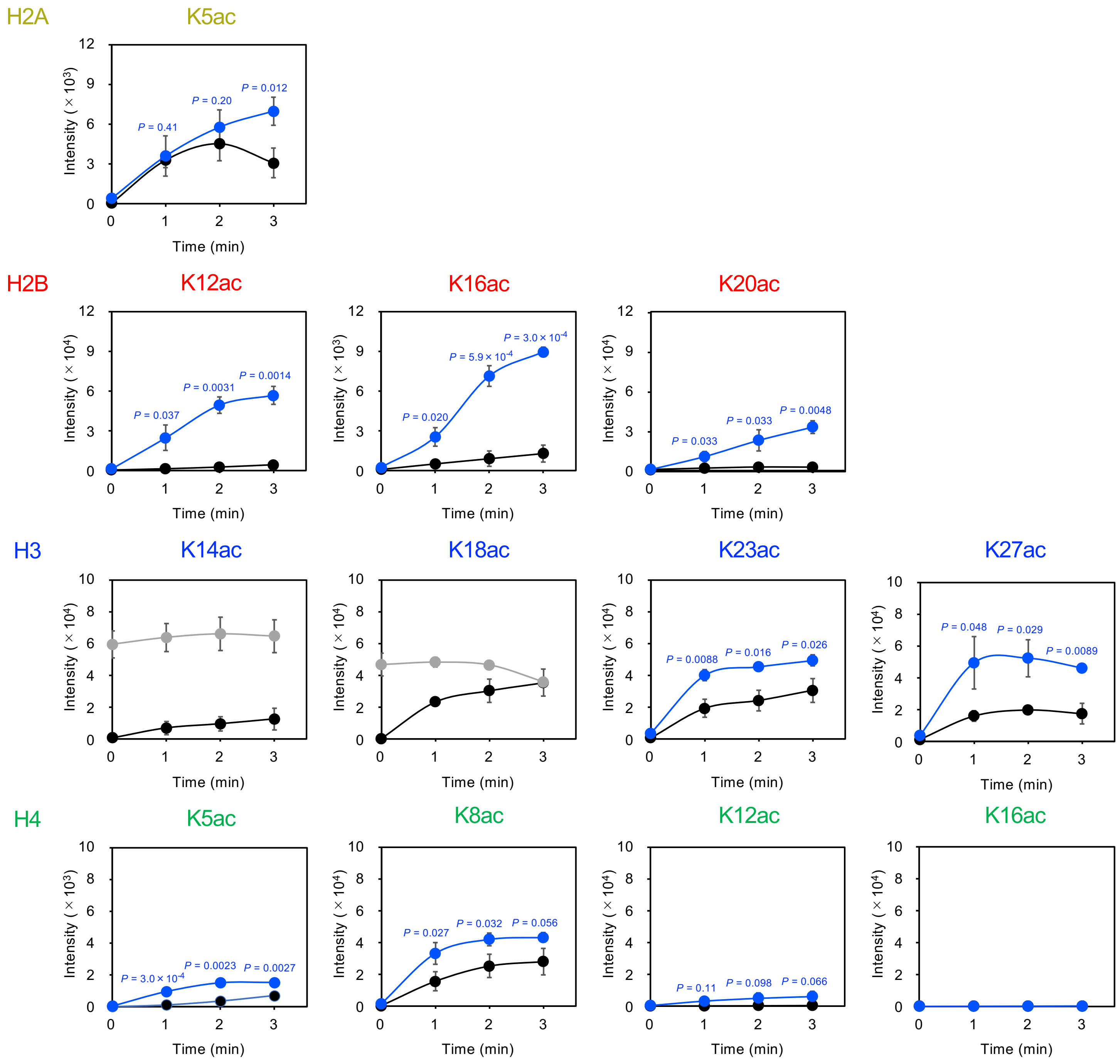

Supplementary Figure 18 In vitro acetyltransferase activity of p300$_{\mathrm{BRPHZT}}$ toward an H3-di-acetylated nucleosome. Residue-specific histone acetylation detected by immunoblotting for each histone species. The position of acetylation is shown above each panel. Black and blue lines indicate the unmodified- and the H3K14/K18-acetylated nucleosomes as substrates (1 µM), respectively. For pre-acetylated H3K14ac and H3K18ac residues, data with the H3K14/K18-acetylated nucleosome as substrate are shown as gray lines. The x-axis indicates the time course after the reaction in the presence of 1 µM p300$_{\mathrm{BRPHZT}}$ and 10 µM acetyl-CoA. The y-axis indicates the immunoblotting signal intensity. Data are mean ± SD from three independent experiments. *P*-value was calculated by a two-sample one-sided Welch's *t*-test for each time point. The alternative hypothesis is that the acetylated nucleosome is more acetylated by p300$_{\mathrm{BRPHZT}}$ than the unmodified nucleosome.

Supplementary Figure 19

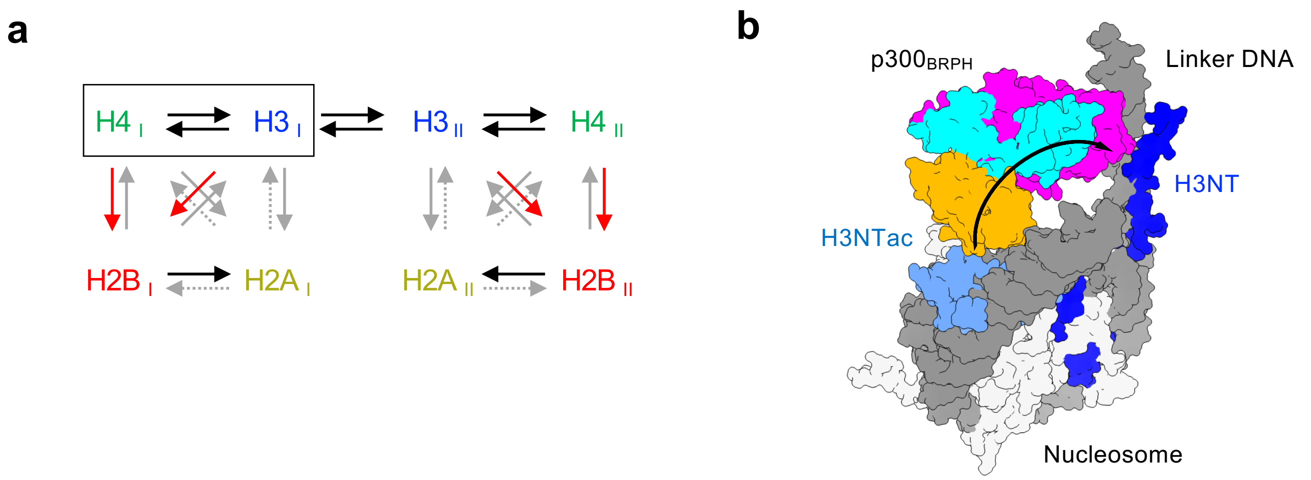

Supplementary Figure 19 Flow of acetylation information propagated by p300. a Summary of read/write flow of histone N-terminal tail acetylation by p300. Each of the four pairs of histones in the nucleosome is shown schematically as I and II. Arrows indicate the read → write direction of histone acetylation and are classified as follows: red, high signal-to-noise ratio signaling; black, moderate signaling; gray, low or no signaling; dotted gray, not determined. The H3-H4 dimer, which could be derived from the parental histone octamer, is schematically enclosed. **b** Hypothetical model in which $p300_{BRPH}$ reads/writes Kac between the H3NT pair. $p300_{BRPH}$ can bind to the nucleosome having linker DNA at both ends, with its bromodomain reading one of a pair of H3 N-terminal tails and its catalytic center, the histone acetyltransferase domain, simultaneously writing Kac to the other H3 N-terminal tail. Color code of $p300_{BRPH}$: orange, bromodomain; cyan, the RING and PHD zinc-fingers; magenta, histone acetyltransferase domain. Nucleosomes, linker DNA, and histone H3 are colored light gray, dark gray, and blue (one pale blue and the other dark blue), respectively. The black arrow indicates read → write direction.

Supplementary Figure 20

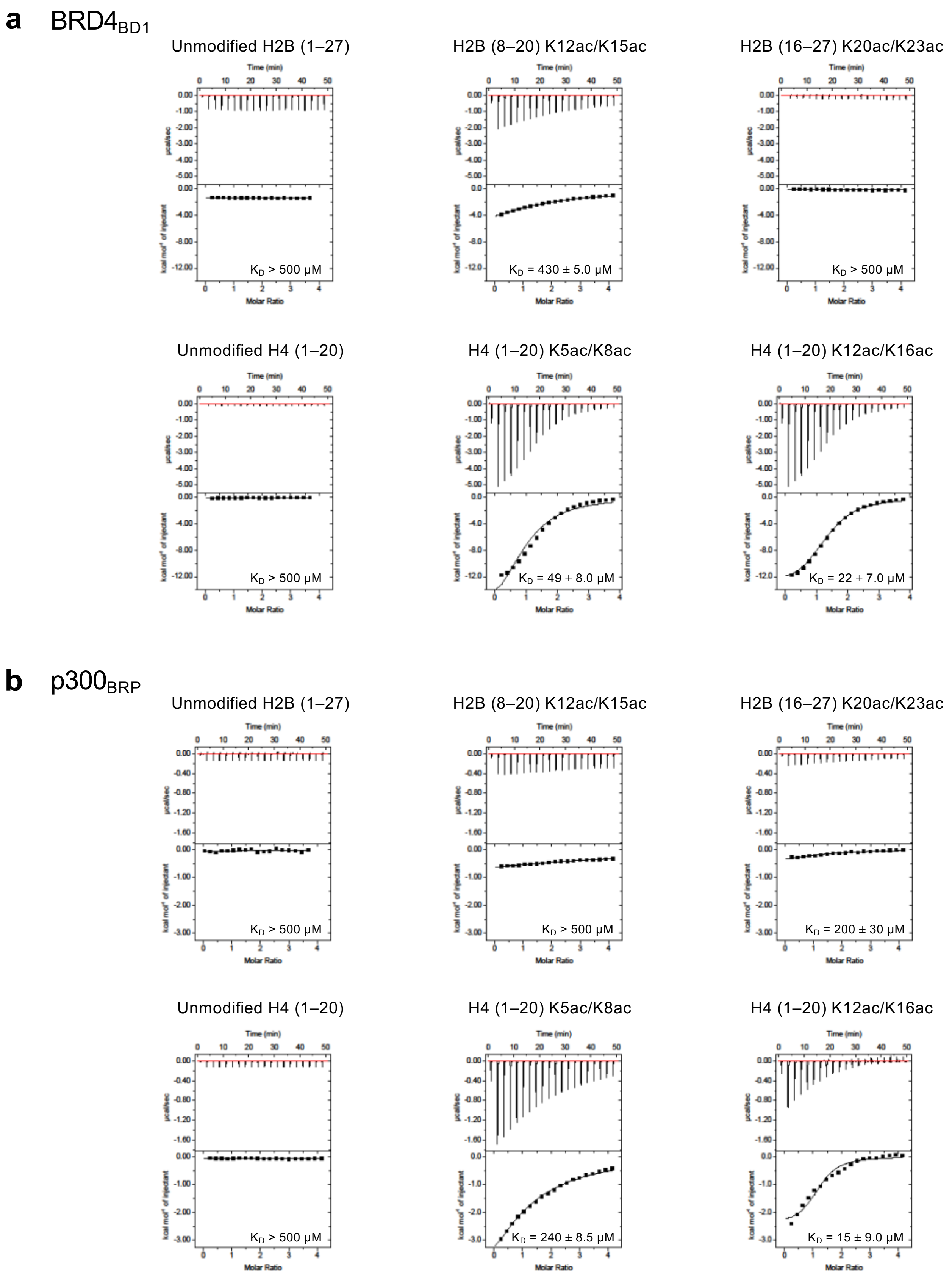

Supplementary Figure 20 Binding analysis between bromodomains and histone peptides measured by isothermal titration calorimetry. **a** Representative thermograms (top) and fitted binding curves of the isotherms (bottom) between $BRD4_{BD1}$ (residues 44–168) and histone peptides. **b** Representative thermograms (top) and fitted binding curves of the isotherms (bottom) between $p300_{BRP}$ (residues 1048–1282) and histone peptides. Histone type, N-terminal tail length, and acetylated lysine position are shown above each panel. K_D values are shown within each panel. Data are mean ± SEM. Each experiment was repeated independently twice with consistency.

Supplementary Figure 21

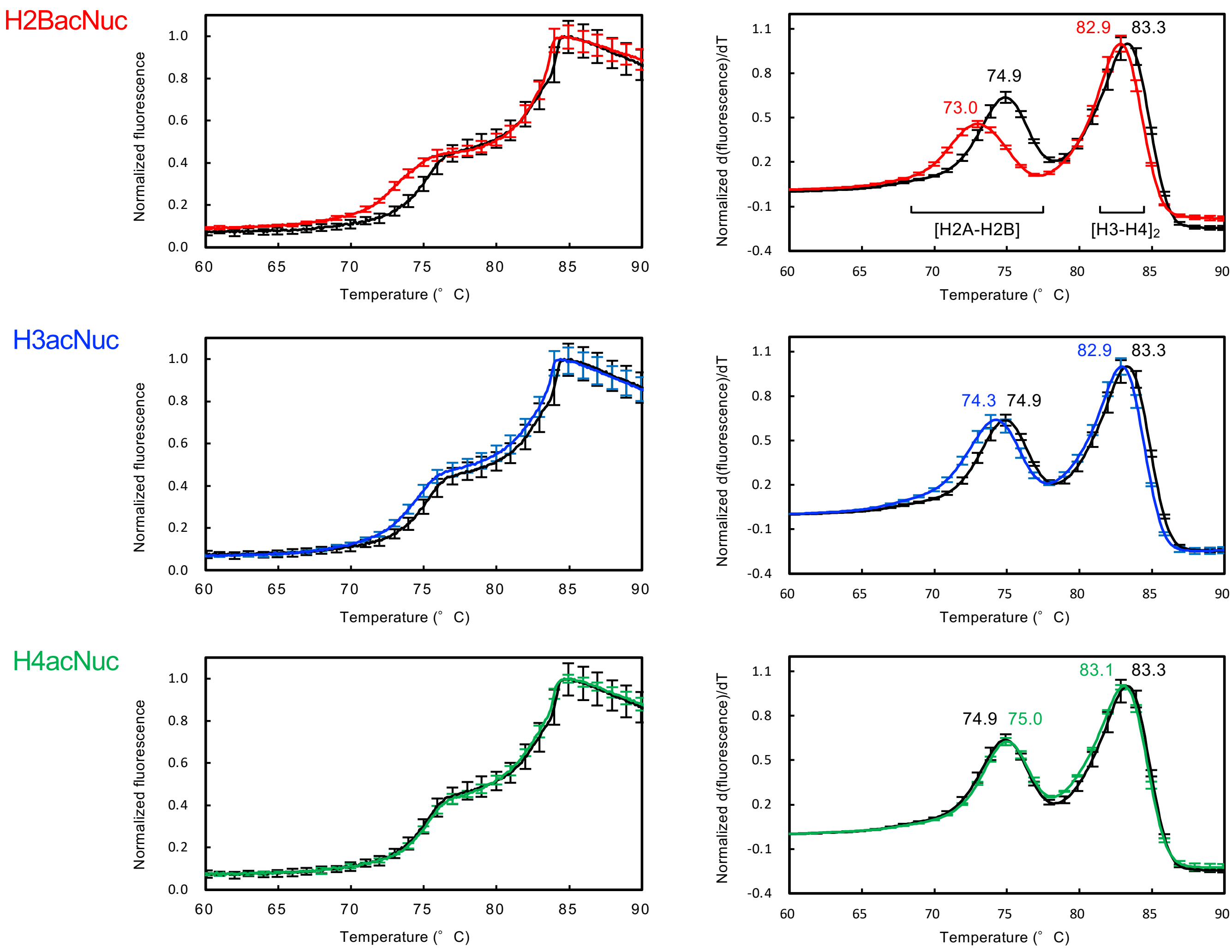

Supplementary Figure 21 Thermal stability assay of acetylated nucleosomes. Mean values of the thermal denaturation curves from 60.0 to 90.0 °C for fluorescence intensity (left) and derivative fluorescence intensity (right) are plotted. The black line indicates the unmodified nucleosome. The colored line indicates residue-specific acetylated nucleosome in the following color code: red, H2BK12/K15/K20/K23-acetylated; blue, H3K14/K18-acetylated; and green, H4K12/K16-acetylated. In the right panels, melting temperature (T_m) is shown at each peak. In the top right panel, the temperature at which the H2A-H2B dimer or the H3-H4 tetramer dissociates from the nucleosome is shown at the bottom. Data are mean ± SD from three independent experiments.

Supplementary Figure 22

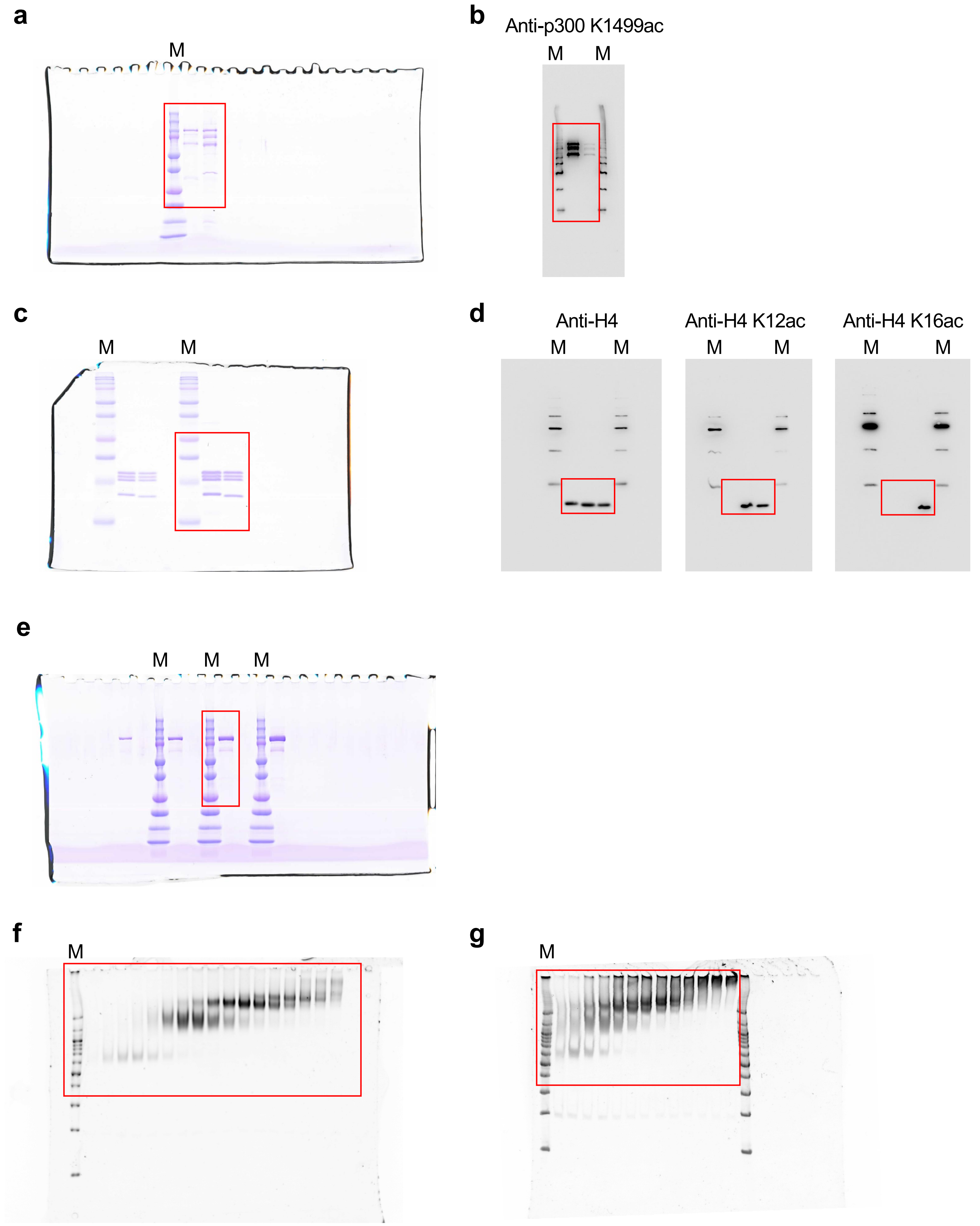

Supplementary Figure 22 (continued)

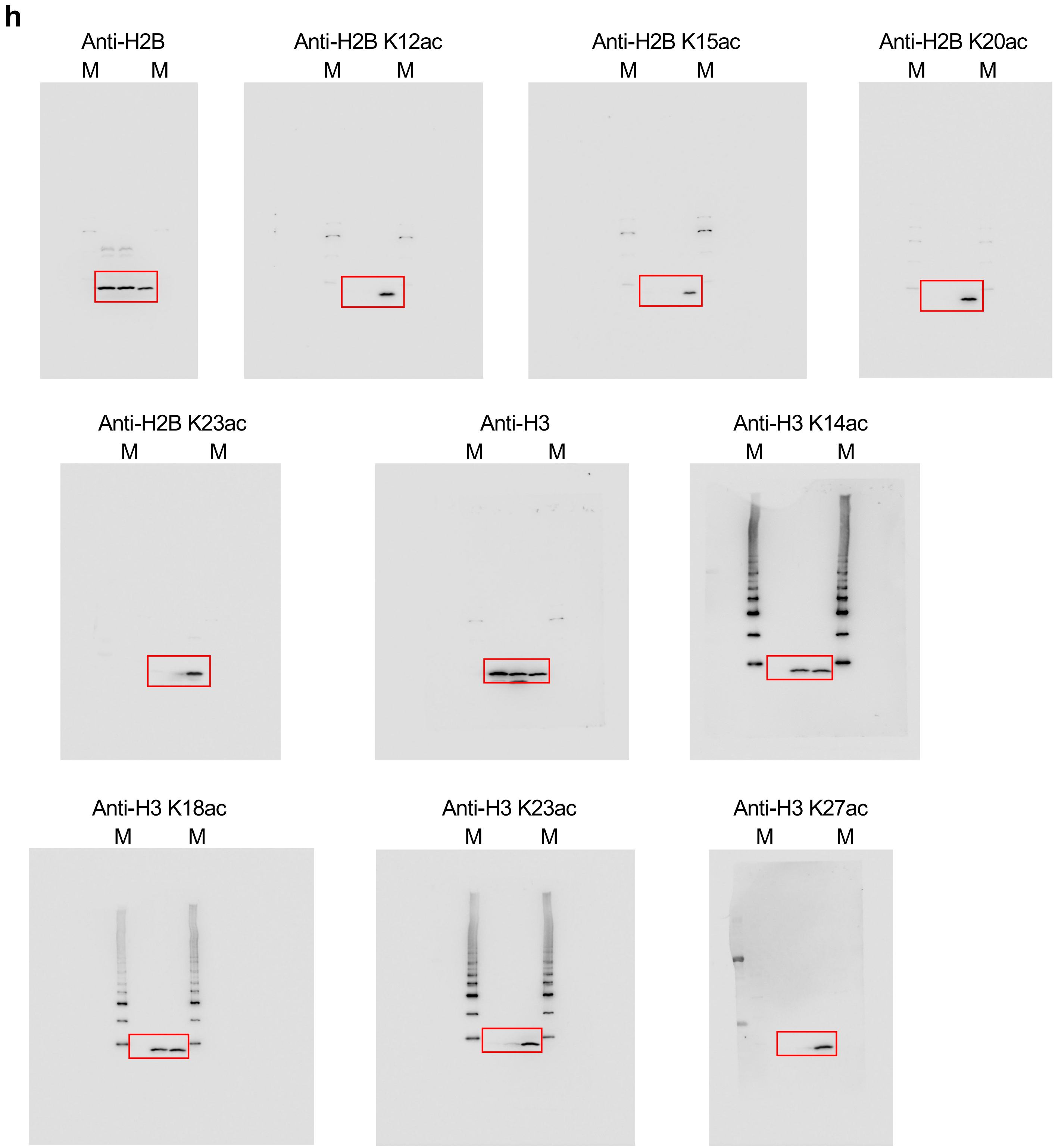

Supplementary Figure 22 Source data for gel images. The cropped images correspond to the source data as follows. **a** Supplementary Fig. 1b. **b** Supplementary Fig. 1c. **c** Supplementary Fig. 1d. **d** Supplementary Fig. 1e. **e** Supplementary Fig. 1f. **f** Supplementary Fig. 5a. **g** Supplementary Fig. 8a. **h** Supplementary Fig. 16a. M: molecular weight marker.

Supplementary Table 1 Acetylated (ac) peptides of $p300_{BRPHZT}$ and their acetylation sites identified by mass spectrometry

Annotated Sequence	Modifications	Modifications in Master Proteins	# Missed Cleavages	Theo. MH+ [Da]	Ions Score (by Search Engine): Mascot	Positions in Proteins	Confidence*
[K].ELEQEEEERKREENTSNESTDVTKGDSK.[N]	1xKac [K24]	Q09472 1xKac [K1542]	4	3338.5102	52	[1519-1546]	High
[K].REENTSNESTDVTKGDSK.[N]	1xKac [K14]	Q09472 1xKac [K1542]	2	2038.9160	74	[1529-1546]	High
[K].REENTSNESTDVTKGDSKNAK.[K]	2xKac [K14; K18]	Q09472 2xKac [K1542; K1546]	3	2394.1015	70	[1529-1549]	High
[K].REENTSNESTDVTKGDSKNAKK.[K]	3xKac [K14; K18; K]	Q09472 3xKac [K1542; K1546; K]	4	2564.2071	76	[1529-1550]	High
[K].GDSKNAKKK.[N]	3xKac [K4; K7; K8]	Q09472 3xKac [K1546; K1549; K1550]	3	1101.5899	45	[1543-1551]	High
[K].KNNKKTSKNK.[S]	4xKac [K1; K4; K5; K8]	Q09472 4xKac [K1551; K1554; K1555; K1558]	4	1357.7434	67	[1551-1560]	High
[K].NNKKTSKNK.[S]	3xKac [K3; K4; K7]	Q09472 3xKac [K1554; K1555; K1558]	3	1187.6379	38	[1552-1560]	High
[K].NNKKTSKNKSSLSR.[G]	4xKac [K3; K4; K7; K9]	Q09472 4xKac [K1554; K1555; K1558; K1560]	4	1759.9297	95	[1552-1565]	High
[K].KTSKNKSSLSR.[G]	3xKac [K1; K4; K6]	Q09472 3xKac [K1555; K1558; K1560]	3	1361.7383	79	[1555-1565]	High
[K].KTSKNKSSLSRGNK.[K]	3xKac [K1; K4; K6]	Q09472 3xKac [K1555; K1558; K1560]	4	1660.8977	39	[1555-1568]	High
[K].TSKNKSSLSR.[G]	2xKac [K3; K5]	Q09472 2xKac [K1558; K1560]	2	1191.6328	60	[1556-1565]	High
[K].TSKNKSSLSRGNK.[K]	2xKac [K3; K5]	Q09472 2xKac [K1558; K1560]	3	1490.7922	43	[1556-1568]	High
[K].NKSSLSRGNK.[K]	1xKac [K2]	Q09472 1xKac [K1560]	2	1132.6069	38	[1559-1568]	High
[K].NKSSLSRGNKK.[K]	2xKac [K2; K10]	Q09472 2xKac [K1560; K1568]	3	1302.7124	46	[1559-1569]	High

*High confidence: with a false discovery rate threshold of 1% using Proteome Discoverer 3.0.

Supplementary Table 2 Acetylated (ac) peptides of p300$_{BRPHZT}$-reacted histone N-terminal tails and their acetylation sites identified by mass spectrometry

Histone	Sequence	Modifications	Modifications in Master Proteins	Positions in Histones	H4K12ac+K16ac/unmod		
					0 min	1 min	3 min
H2A	[-].SGRGKQGGKARAK.[A]	2xKac [K5; K9]	H2A 2xKac [K5; K9]	H2A [1-13]	ND	3.36 ± 0.33	2.16 ± 0.20
H2B	[-].gPEPAKSAPAPK.[K]	1xKac [K6]	H2B 1xKac [K5]	H2B [1-11]	0.719 ± 0.090	4.97 ± 0.40	3.64 ± 0.28
	[K].SAPAPKKGSKKAVTK.[A]	4xKac [K6; K7; K10; K11]	H2B 4xKac [K11; K12; K15; K16]	H2B [6-20]	ND	++	++
	[K].SAPAPKKGSKK.[A]	3xKac [K6; K; K]	H2B 3xKac [K11; K; K]	H2B [6-16]	ND	10.2 ± 1.5	15.4 ± 1.1
	[K].SAPAPKKGSK.[K]	2xKac [K6; K7]	H2B 2xKac [K11; K12]	H2B [6-15]	ND	4.31 ± 0.40	5.09 ± 0.37
	[K].KGSKKAVTKAQK.[K]	4xKac [K1; K4; K5; K9]	H2B 4xKac [K12; K15; K16; K20]	H2B [12-23]	ND	12.1 ± 1.9	20.8 ± 2.1
	[K].KGSKKAVTK.[A]	3xKac [K1; K4; K5]	H2B 3xKac [K12; K15; K16]	H2B [12-20]	ND	22.2 ± 2.2	21.2 ± 1.6
	[K].GSKKAVTKAQK.[K]	3xKac [K3; K4; K8]	H2B 3xKac [K15; K16; K20]	H2B [13-23]	0	45.8 ± 4.4	68.2 ± 8.2
	[K].GSKKAVTK.[A]	2xKac [K3; K4]	H2B 2xKac [K15; K16]	H2B [13-20]	ND	11.7 ± 1.7	7.29 ± 0.58
	[K].AVTKAQKK.[D]	2xKac [K4; K]	H2B 2xKac [K20; K]	H2B [17-24]	ND	31.2 ± 2.9	29.1 ± 3.3
	[K].AVTKAQK.[K]	1xKac [K4]	H2B 2xKac [K20]	H2B [17-23]	1.38 ± 0.20	14.8 ± 1.3	14.8 ± 0.98
H3	[K].STGGKAPRKQLATKAAR.[K]	3xKac [K5; K9; K14]	H3.1 3xKac [K14; K18; K23]	H3.1 [10-26]	ND	10.5 ± 0.79	7.38 ± 0.78
	[K].STGGKAPRKQLATK.[A]	2xKac [K5; K9]	H3.1 2xKac [K14; K18]	H3.1 [10-23]	ND	6.62 ± 0.48	4.23 ± 0.36
	[K].STGGKAPRKQLATK.[A]	3xKac [K5; K9; K14]	H3.1 3xKac [K14; K18; K23]	H3.1 [10-23]	ND	++	6.21 ± 1.1
	[K].STGGKAPRK.[Q]	1xKac [K5]	H3.1 1xKac [K14]	H3.1 [10-18]	0.846 ± 0.045	0.724 ± 0.024	0.624 ± 0.039
	[K].APRKQLATKAARK.[S]	2xKac [K4; K9]	H3.1 2xKac [K18; K23]	H3.1 [15-27]	ND	1.52 ± 0.94	2.69 ± 0.17
	[K].APRKQLATK.[A]	1xKac [K4]	H3.1 1xKac [K18]	H3.1 [15-23]	0.103 ± 0.018	1.85 ± 0.086	1.30 ± 0.10
	[R].KQLATKAARKSAPATGGVK.[K]	3xKac [K1; K6; K10]	H3.1 3xKac [K18; K23; K27]	H3.1 [18-36]	ND	9.91 ± 1.2	8.41 ± 0.54
	[R].KQLATKAARK.[S]	2xKac [K1; K6]	H3.1 2xKac [K18; K23]	H3.1 [18-27]	ND	6.71 ± 0.57	6.11 ± 0.44
	[R].KQLATKAAR.[K]	2xKac [K1; K6]	H3.1 2xKac [K18; K23]	H3.1 [18-26]	ND	11.4 ± 0.92	6.69 ± 0.49
	[K].QLATKAARKSAPATGGVK.[K]	2xKac [K5; K9]; Q->pyro-Glu [N-Term]	H3.1 2xKac [K23; K27]	H3.1 [19-36]	ND	7.41 ± 0.91	2.54 ± 0.17
	[K].QLATKAARKSAPATGGVK.[K]	2xKac [K5; K9]	H3.1 2xKac [K23; K27]	H3.1 [19-36]	ND	6.44 ± 0.44	2.79 ± 0.20
	[K].QLATKAARK.[S]	1xKac [K5]	H3.1 1xKac [K23]	H3.1 [19-27]	0.0675 ± 0.0048	1.69 ± 0.096	1.06 ± 0.073
	[K].AARKSAPATGGVK.[K]	1xKac [K4]	H3.1 1xKac [K27]	H3.1 [24-36]	0.0748 ± 0.0060	3.51 ± 0.28	2.20 ± 0.24
	[R].KSAPATGGVK.[K]	1xKac [K1]	H3.1 1xKac [K27]	H3.1 [27-36]	0	3.29 ± 0.34	3.07 ± 0.42
	[K].SAPATGGVKK.[P]	1xKac [K9]	H3.1 1xKac [K36]	H3.1 [28-37]	0.0699 ± 0.0038	0.343 ± 0.019	1.04 ± 0.60
H4	[-].SGRGKGGKGLGK.[G]	2xKac [K5; K8]	H4 2xKac [K5; K8]	H4 [1-12]	ND	0	0
	[K].GGKGLGKGGAK.[R]	2xKac [K3; K7]	H4 2xKac [K8; K12]	H4 [6-16]	ND	0.418 ± 0.034	0.0916 ± 0.0092
	[K].GGKGLGKGGAK.[R]	3xKac [K3; K7; K11]	H4 3xKac [K8; K12; K16]	H4 [6-16]	++	++	++
	[K].GGKGLGK.[G]	1xKac [K3]	H4 1xKac [K8]	H4 [6-12]	0.175 ± 0.012	0.00609 ± 0.00029	0.00353 ± 0.00017
	[K].GLGKGGAKRHRK.[V]	2xKac [K4; K8]	H4 2xKac [K12; K16]	H4 [9-20]	++	++	++
	[K].GLGKGGAKRHR.[K]	1xKac [K8]	H4 1xKac [K16]	H4 [9-19]	++	++	++
	[K].GLGKGGAKRHR.[K]	2xKac [K4; K8]	H4 2xKac [K12; K16]	H4 [9-19]	++	++	++
	[K].GLGKGGAKR.[H]	1xKac [K8]	H4 1xKac [K16]	H4 [9-17]	++	++	++
	[K].GLGKGGAKR.[H]	2xKac [K4; K8]	H4 2xKac [K12; K16]	H4 [9-17]	++	++	2710 ± 230
	[K].GLGKGGAK.[R]	1xKac [K4]	H4 1xKac [K12]	H4 [9-16]	3.30 ± 0.23	0.547 ± 0.030	0.331 ± 0.019
	[K].GLGKGGAK.[R]	2xKac [K4; K8]	H4 2xKac [K12; K16]	H4 [9-16]	++	++	++

Data are mean ± SEM from three technical replicates. ND, not determined. ++, Signal was detected in H4K12ac+K16ac with no signal in the unmodified nucleosome.

Supplementary Table 3 Cryo-electron microscopy data collection, refinement, and validation statistics

	#1 p300$_{H2B}$·H4acNuc (EMD-34588, PDB 8HAG)	#2 p300$_{H3\text{-}I}$·H4acNuc (EMD-34589, PDB 8HAH)	#3 p300$_{H2A}$·H4acNuc (EMD-34590)	#4 p300$_{H2B}$·H4acNuc (EMD-34591, PDB 8HAI)	#5 p300$_{H3\text{-}I}$·H4acNuc (EMD-34592, PDB 8HAJ)	#6 p300$_{H3\text{-}II}$·H4acNuc (EMD-34593)	#7 p300$_{H2A}$·H4acNuc (EMD-34594, PDB 8HAK)
**Data collection and processing**							
Microscope	Titan Krios G4	Titan Krios G4	Titan Krios G4	Tecnai Arctica	Tecnai Arctica	Tecnai Arctica	Tecnai Arctica
Magnification	105,000	105,000	105,000	23,500	23,500	23,500	23,500
Voltage (kV)	300	300	300	200	200	200	200
Electron exposure (e–/Å^2)	50	50	50	50	50	50	50
Defocus range (μm)	-0.8– -2.0	-0.8– -2.0	-0.8– -2.0	-0.9– -1.7	-0.9– -1.7	-0.9– -1.7	-0.9– -1.7
Pixel size (Å)	0.829	0.829	0.829	1.47	1.47	1.47	1.47
Symmetry imposed	C1	C1	C1	C1	C1	C1	C1
Initial particle images (no.)	3,854,288	3,854,288	3,854,288	12,122,750	12,122,750	12,122,750	12,122,750
Final particle images (no.)	113,423	74,692	25,558	157,198	156,863	61,319	89,085
Map resolution (Å)	3.2	3.9	3.9	4.7	4.8	6.9	4.5
FSC threshold	0.143	0.143	0.143	0.143	0.143	0.143	0.143
Map resolution range (Å)	3.1–8.4	3.5–10.6	3.6–10.3	4.3–11.3	3.1–9.3	3.1–9.3	4.1–12.7
**Refinement**							
Initial model used (PDB code)	1KX3, 6GYR	1KX3, 6GYR		1KX3, 6GYR	1KX3, 6GYR		1KX3, 6GYR
Model resolution (Å)	3.6	8.6		6.0	6.7		6.0
FSC threshold	0.5	0.5		0.5	0.5		0.5
Map sharpening *B* factor (Å^2)	-10	-30		-180	-119		-90
Model-map scores							
CC (mask)	0.78	0.62		0.72	0.76		0.75
CC (box)	0.85	0.83		0.83	0.88		0.87
CC (peaks)	0.70	0.42		0.68	0.69		0.69
CC (volume)	0.76	0.56		0.73	0.74		0.74
Model composition							
Non-hydrogen atoms	16,367	17,096		16,547	17,214		16,342
Protein residues	1284	1287		1310	1305		1285
Nucleotides	294	328		294	326		292
R.m.s. deviations							
Bond lengths (Å)	0.003	0.003		0.003	0.003		0.003
Bond angles (°)	0.547	0.539		0.566	0.592		0.641
**Validation**							
MolProbity score	1.54	1.59		1.55	1.63		1.67
Clashscore	10.55	8.69		10.75	11.31		12.75
Poor rotamers (%)	0.00	0.00		0.00	0.00		0.00
Ramachandran plot							
Favored (%)	98.33	97.39		98.52	98.04		97.77
Allowed (%)	1.67	2.61		1.48	1.96		2.33
Disallowed (%)	0.00	0.00		0.00	0.00		0.00

Supplementary Table 3 (continued) Cryo-electron microscopy data collection, refinement, and validation statistics

	#8 CBP$_{H2B}$· H4acNuc (EMD-34595, PDB 8HAL)	#9 CBP$_{H3-I}$· H4acNuc (EMD-34596, PDB 8HAM)	#10 CBP$_{H3-II}$· H4acNuc (EMD-34597, PDB 8HAN)
**Data collection and processing**			
Microscope	Tecnai Arctica	Tecnai Arctica	Tecnai Arctica
Magnification	23,500	23,500	23,500
Voltage (kV)	200	200	200
Electron exposure (e–/Å^2)	50	50	50
Defocus range (μm)	-0.9– -1.7	-0.9– -1.7	-0.9– -1.7
Pixel size (Å)	1.47	1.47	1.47
Symmetry imposed	C1	C1	C1
Initial particle images (no.)	12,594,925	12,594,925	12,594,925
Final particle images (no.)	119,741	90,318	207,123
Map resolution (Å)	4.4	4.5	4.2
FSC threshold	0.143	0.143	0.143
Map resolution range (Å)	4.0–8.7	4.1–12.1	3.9–12.4
**Refinement**			
Initial model used (PDB code)	1KX3, 5U7G	1KX3, 5U7G	1KX3, 5U7G
Model resolution (Å)	6.1	4.5	6.6
FSC threshold	0.5	0.5	0.5
Map sharpening *B* factor (Å^2)	-50	-100	-60
Model-map scores			
CC (mask)	0.73	0.77	0.72
CC (box)	0.85	0.84	0.83
CC (peaks)	0.67	0.72	0.67
CC (volume)	0.73	0.78	0.72
Model composition			
Non-hydrogen atoms	16,585	17,177	16,180
Protein residues	1292	1288	1261
Nucleotides	302	330	294
R.m.s. deviations			
Bond lengths (Å)	0.003	0.003	0.003
Bond angles (°)	0.619	0.555	0.520
**Validation**			
MolProbity score	1.60	1.50	1.45
Clashscore	12.30	9.55	8.34
Poor rotamers (%)	0.00	0.00	0.00
Ramachandran plot			
Favored (%)	98.26	98.57	98.86
Allowed (%)	1.74	1.43	1.14
Disallowed (%)	0.00	0.00	0.00

Supplementary Table 4 Binding analysis between p300 bromodomain and nucleosomes, measured by microscale thermophoresis

$p300_{BRP}$	Nucleosome	CBP30	$K_{1/2}$ (nM)
Wild-type	Unmodified	none	2.2 ± 0.51
	H4K12/K16-acetylated	none	0.35 ± 0.10
	H4K12/K16-acetylated	10 μM	1.2 ± 0.20
4A	Unmodified	none	7.3 ± 1.6
	H4K12/K16-acetylated	none	1.8 ± 0.22
	H4K12/K16-acetylated	10 μM	3.5 ± 1.6

Data are mean ± SEM from three independent experiments. $p300_{BRP}$, Wild-type of the BD-RING-PHD domain (residues 1048–1282) of human p300. 4A, the BD-RING-PHD domain (1048–1282) with mutations of R1133A, K1134A, R1137A, and K1140A.

Supplementary Table 5 Dissociation constants between bromodomains (BD) and histone peptides measured by isothermal titration calorimetry

BD	Histone peptide (residues)	Modification	K_D (μM)
$BRD4_{BD1}$	H2B (1–27)	none	ND
	H2B (8–20)	K12ac/K15ac	430 ± 5.0
	H2B (16–27)	K20ac/K23ac	ND
	H4 (1–20)	none	ND
	H4 (1–20)	K5ac/K8ac	49 ± 8.0
	H4 (1–20)	K12ac/K16ac	22 ± 7.0
$p300_{BRP}$	H2B (1–27)	none	ND
	H2B (8–20)	K12ac/K15ac	ND
	H2B (16–27)	K20ac/K23ac	200 ± 30
	H4 (1–20)	none	ND
	H4 (1–20)	K5ac/K8ac	240 ± 8.5
	H4 (1–20)	K12ac/K16ac	15 ± 9.0

ND, not determined because K_D is greater than 500 μM. Data are mean ± SEM from representative thermograms. Experiments were repeated independently twice with consistency. $BRD4_{BD1}$, the N-terminal BD (residues 44–168) of human BRD4. $p300_{BRP}$, the BD-RING-PHD domain (residues 1048–1282) of human p300.
