# Supplementary material for: Epigenetic mechanisms to propagate histone acetylation by p300/CBP
Source: Nat Commun. 2023 Jul 17;14:4103. doi: 10.1038/s41467-023-39735-4 (PMC10352329; doi:10.1038/s41467-023-39735-4)
Supplement: Supplementary file 3 — Reporting Summary [file 41467_2023_39735_MOESM3_ESM.pdf]

nature portfolio

Corresponding author(s): Takashi Umehara

Last updated by author(s): May 17, 2023

Reporting Summary

Nature Portfolio wishes to improve the reproducibility of the work that we publish. This form provides structure for consistency and transparency in reporting. For further information on Nature Portfolio policies, see our Editorial Policies and the Editorial Policy Checklist.

Statistics

For all statistical analyses, confirm that the following items are present in the figure legend, table legend, main text, or Methods section.

n/a	Confirmed	
☐	☒	The exact sample size (*n*) for each experimental group/condition, given as a discrete number and unit of measurement
☐	☒	A statement on whether measurements were taken from distinct samples or whether the same sample was measured repeatedly
☐	☒	The statistical test(s) used AND whether they are one- or two-sided<br>*Only common tests should be described solely by name; describe more complex techniques in the Methods section.*
☒	☐	A description of all covariates tested
☒	☐	A description of any assumptions or corrections, such as tests of normality and adjustment for multiple comparisons
☐	☒	A full description of the statistical parameters including central tendency (e.g. means) or other basic estimates (e.g. regression coefficient) AND variation (e.g. standard deviation) or associated estimates of uncertainty (e.g. confidence intervals)
☐	☒	For null hypothesis testing, the test statistic (e.g. *F*, *t*, *r*) with confidence intervals, effect sizes, degrees of freedom and *P* value noted<br>*Give P values as exact values whenever suitable.*
☒	☐	For Bayesian analysis, information on the choice of priors and Markov chain Monte Carlo settings
☒	☐	For hierarchical and complex designs, identification of the appropriate level for tests and full reporting of outcomes
☒	☐	Estimates of effect sizes (e.g. Cohen's *d*, Pearson's *r*), indicating how they were calculated

Our web collection on statistics for biologists contains articles on many of the points above.

Software and code

Policy information about availability of computer code

Data collection	SerialEM (v 3.8), EPU (v2.9)
Data analysis	ImageJ 1.53, Microsoft 365 (Excel), RELION-3.1, MotionCor2, CTFFIND-4.1, crYOLO v1.7.6, Chimera v1.15, AlphaFold v2.2.2, Coot-0.9.8.1, ISOLDE 1.4, PHENIX v1.18.2 (Phenix Real-space refinement, MolProbity), ChimeraX v1.4, PyMOL v2.5, MO.Affinity analysis v2.3, Origin 7 SR4 v7.0552, Applied Biosystems Protein Thermal Shift Software v1.2 (Build Information: Quente_FC1_B2_20130625094457, Algorithm version: v1.4), MASCOT 2.8, Proteome Discoverer 3.0, ClustalW 2.1, ESPript 3.0

For manuscripts utilizing custom algorithms or software that are central to the research but not yet described in published literature, software must be made available to editors and reviewers. We strongly encourage code deposition in a community repository (e.g. GitHub). See the Nature Portfolio guidelines for submitting code & software for further information.

Data

Policy information about availability of data

All manuscripts must include a data availability statement. This statement should provide the following information, where applicable:

- Accession codes, unique identifiers, or web links for publicly available datasets
- A description of any restrictions on data availability
- For clinical datasets or third party data, please ensure that the statement adheres to our policy

The cryo-EM density maps have been deposited in the Electron Microscopy Data Bank (EMDB, www.ebi.ac.uk/pdbe/emdb/) under the accession codes EMD-34588, EMD-34589, EMD-34590, EMD-34591, EMD-34592, EMD-34593, EMD-34594, EMD-34595, EMD-34596, and EMD-34597. The atomic coordinates have been

deposited in the Protein Data Bank (PDB, www.rcsb.org) under the accession codes 8HAG, 8HAH, 8HAI, 8HAJ, 8HAK, 8HAL, 8HAM, and 8HAN. Structural models used in this study can be found in PDB under the accession codes 1KX3, 5U7G, 6GYR, and 7W9V, and AlphaFold Protein Structure Database under the accession code AF-Q92793-F1. The mass spectrometry proteomics data have been deposited with the ProteomeXchange Consortium via the PRIDE partner repository with the dataset identifier PXD040835.

Human research participants

Policy information about studies involving human research participants and Sex and Gender in Research.

Reporting on sex and gender	n/a
Population characteristics	n/a
Recruitment	n/a
Ethics oversight	n/a

Note that full information on the approval of the study protocol must also be provided in the manuscript.

Field-specific reporting

Please select the one below that is the best fit for your research. If you are not sure, read the appropriate sections before making your selection.

☒ Life sciences ☐ Behavioural & social sciences ☐ Ecological, evolutionary & environmental sciences

For a reference copy of the document with all sections, see nature.com/documents/nr-reporting-summary-flat.pdf

Life sciences study design

All studies must disclose on these points even when the disclosure is negative.

Sample size	No statistical methods were used to determine sample size. The size of the cryo-EM data was determined based on previous studies (PMIDs: 33361816, 33972509, and 34301908). By using the RELION-3.1 program, 25,558–207,123 particles were finally selected to reconstitute the 3.2–6.9 Å resolution maps, which was sufficient to build atomic models of the p300/CBP-nucleosome complexes. The details of cryo-EM datasets including sample sizes are provided in Supplementary Information. Acetyltransferase assays, microscale thermophoresis analysis, and nucleosome thermostability assays were repeated independently three times. Mass spectrometric analysis was technically repeated three times. Polyacrylamide gel electrophoresis of prepared samples, electrophoretic mobility shift assays, cryo-electron micrograph acquisition, and isothermal titration calorimetry experiments were repeated independently twice.
Data exclusions	The initial cryo-EM images were screened manually to exclude those with low contrast, thick ice, or severe ice contaminations, which is a standard procedure for cryo-EM data processing. No biochemical data were excluded.
Replication	Acetyltransferase assays, microscale thermophoresis analysis, and nucleosome thermostability assays were repeated independently three times. Mass spectrometric analysis was technically repeated three times. Polyacrylamide gel electrophoresis of prepared samples, electrophoretic mobility shift assays, cryo-electron micrograph acquisition, and isothermal titration calorimetry experiments were repeated independently twice. All attempts at replication were successful.
Randomization	Randomization is not relevant to this study since it does not include animals or human participants. For cryo-EM structure determination, the data were randomly divided into two sets by the RELION-3.1 program.
Blinding	Not blinded. Because these experiments needed specialized handling techniques, the number of well-trained researchers was limited.

Reporting for specific materials, systems and methods

We require information from authors about some types of materials, experimental systems and methods used in many studies. Here, indicate whether each material, system or method listed is relevant to your study. If you are not sure if a list item applies to your research, read the appropriate section before selecting a response.

Materials & experimental systems

n/a	Involved in the study
☐	☒ Antibodies
☐	☒ Eukaryotic cell lines
☒	☐ Palaeontology and archaeology
☒	☐ Animals and other organisms
☒	☐ Clinical data
☒	☐ Dual use research of concern

Methods

n/a	Involved in the study
☒	☐ ChIP-seq
☒	☐ Flow cytometry
☒	☐ MRI-based neuroimaging

Antibodies

Antibodies used	anti-H2AK5ac (Abcam, ab45152, 1/3,000), anti-H2B (Cell Signaling, 12364, 1/1,000), anti-H2BK12ac (Abcam, ab40883, 1/500), anti-H2BK15ac (Abcam, ab62335, 1/500), anti-H2BK16ac (Abcam, ab177427, 1/1,000), anti-H2BK20ac (Abcam, ab177430, 1/500), anti-H2BK23ac (Abcam, ab222770, 1/1,000), anti-H3 (Merck, 07-690, 1/3,000), anti-H3K14ac (Merck, 07-353, 1/1,000), anti-H3K18ac (Abcam, ab1191, 1/1,000), anti-H3K23ac (Merck, 07-355, 1/1,000), anti-H3K27ac (Merck, 07-360, 1/3,000), anti-H4 (Abcam, ab10158, 1/1,000), anti-H4K5ac (MABI, MABI0405, 1/500), anti-H4K8ac (MABI, MABI0408, 1/500), anti-H4K12ac (MABI, MABI0412, 1/500), anti-H4K16ac (MABI, MABI0416, 1/500), anti-p300K1499ac (Cell Signaling, 4771, 1/1,000), anti-rabbit-IgG HRP (Cytiva, NA934, 1/10,000), and anti-mouse-IgG HRP (Cytiva, NA931, 1/10,000)
Validation	anti-H2AK5ac (RRID: AB_732911), anti-H2B (RRID: AB_2714167), anti-H2BK12ac (RRID: AB_732915), anti-H2BK15ac (RRID: AB_955894), anti-H2BK23ac (RRID: AB_2716413), anti-H3 (RRID: AB_417398), anti-H3K14ac (RRID: AB_310545), anti-H3K18ac (RRID: AB_298692), anti-H3K23ac (RRID: AB_310546), anti-H3K27ac (RRID: AB_310550), anti-H4 (RRID: AB_296888), anti-H4K5ac (RRID: AB_2793668), anti-H4K8ac (RRID: AB_2793669), anti-H4K12ac (RRID: AB_2793670), anti-H4K16ac (RRID: AB_2793671), anti-p300K1499ac (RRID: AB_2262406), anti-rabbit IgG HRP (RRID: AB_772206), and anti-mouse IgG HRP (RRID: AB_772210).<br>The following antibodies were validated by the manufacturer. Validation data and references are available on the manufacturer's website.<br>anti-H2BK16ac (https://www.abcam.co.jp/products/primary-antibodies/histone-h2b-acetyl-k16-antibody-epr17598-chip-grade-ab177427.html)<br>anti-H2BK20ac (https://www.abcam.co.jp/products/primary-antibodies/histone-h2b-acetyl-k20-antibody-epr859-chip-grade-ab177430.html)

Eukaryotic cell lines

Policy information about cell lines and Sex and Gender in Research

Cell line source(s)	High Five cells (Thermo Fisher Scientific, B85502)
Authentication	The cell line was purchased commercially and not authenticated.
Mycoplasma contamination	Not tested because it was used only for protein expression.
Commonly misidentified lines (See ICLAC register)	No commonly misidentified cell lines were used.
